# Comprehensive functional analysis of the PYL-PP2C-SnRK2s family in *Bletilla striata* reveals that BsPP2C22 and BsPP2C38 interact with BsPYLs and BsSnRK2s in response to multiple abiotic stresses

**DOI:** 10.3389/fpls.2022.963069

**Published:** 2022-08-11

**Authors:** Shuai Liu, Chan Lu, Guanghui Jiang, Ru Zhou, Yuanqing Chang, Shiqiang Wang, Donghao Wang, Junfeng Niu, Zhezhi Wang

**Affiliations:** National Engineering Laboratory for Resource Development of Endangered Crude Drugs in Northwest China, Key Laboratory of the Ministry of Education for Medicinal Resources and Natural Pharmaceutical Chemistry, College of Life Sciences, Shaanxi Normal University, Xi’an, China

**Keywords:** PYL-PP2C-SnRK2s, protein interaction, *Bletilla striata*, abiotic stresses, ABA signaling pathway

## Abstract

As the core regulation network for the abscisic acid (ABA) signaling pathway, the PYL-PP2C-SnRK2s family commonly exists in many species. For this study, a total of 9 BsPYLs, 66 BsPP2Cs, and 7 BsSnRK2s genes were identified based on the genomic databases of *Bletilla striata*, which were classified into 3, 10, and 3 subgroups, respectively. Basic bioinformatics analysis completed, including the physicochemical properties of proteins, gene structures, protein motifs and conserved domains. Multiple *cis*-acting elements related to stress responses and plant growth were found in promoter regions. Further, 73 genes were localized on 16 pseudochromosomes and 29 pairs of paralogous genes were found via intraspecific collinearity analysis. Furthermore, tissue-specific expression was found in different tissues and germination stages. There were two *BsPYLs*, 10 *BsPP2Cs*, and four *BsSnRK2* genes that exhibited a difference in response to multiple abiotic stresses. Moreover, subcellular localization analysis revealed six important proteins BsPP2C22, BsPP2C38, BsPP2C64, BsPYL2, BsPYL8, and BsSnRK2.4 which were localized in the nucleus and plasma membrane. Finally, yeast two-hybrid (Y2H) and bimolecular fluorescence complementation (BiFC) assays suggested that BsPP2C22 and BsPP2C38 could interact with multiple BsPYLs and BsSnRK2s proteins. This study systematically reported on the identification and characterization of the PYL-PP2C-SnRK2s family in *B. striata*, which provided a conceptual basis for deep insights into the functionality of ABA core signal pathways in Orchidaceae.

## Introduction

*Bletilla striata* (Thunb. ex Murray) Reichb. f. (also known as Zilan) is a perennial herb of the *Bletilla* genera in Orchidaceae. It has excellent ornamental qualities, as well as medicinal value via its dried pseudobulb. Previous studies have shown that in addition to being an anti-bacterial and anti-inflammatory, *B. striata* can promote wound-healing due to its multiple medical compositions that include polysaccharides (BSP), bibenzyls, flavonols, and phenanthrenes (Yi et al., 2010; He et al., 2017; Xu et al., 2019; Wang et al., 2020). The content of these compositions is closely related to the growing environment of *B. striata* (Yang et al., 2018; Isah, 2019; Mareri et al., 2022). During the planting process, the yield of medicinal plants was also affected by a variety of abiotic stresses, including low temperature, drought, damage and heavy metal pollution, etc. (Lajayer et al., 2017; González-Villagra et al., 2018; Mareri et al., 2022); additionally, as a typical Orchidaceae plant, its seeds are thin and without endosperm; thus, it is extremely difficult to germinate under natural conditions (Arditti and Ghani, 2013), which are closely related to the abscisic acid (ABA) signal transduction.

Abscisic acid is a key plant hormone involved in multiple biological processes including seed germination and dormancy, seedling growth and development, stress responses, and stomatal aperture (Zhu, 2002; Nambara and Marion-Poll, 2005; Cutler et al., 2010; Chen et al., 2020; Hsu et al., 2021). Since ABA was discovered in the 1960s, the core components of its signaling pathways have been identified over the last 50 years (Cutler et al., 2010; Chen et al., 2020), of which the PYL-PP2C-SnRK2s family of proteins are the main members. The pyrabactin resistance/pyrabactin resistance-like/regulatory components of the abscisic acid receptor (PYR/PYL/RCAR) protein family was identified as a family of ABA receptors in 2009 (Hsu et al., 2021). They belong to a branch of the Bet v superfamily, which is structurally characterized through the presence of the Bet v fold or START domain (Radauer et al., 2008; Yadav et al., 2020). To date, 14 PYL proteins have been identified in *Arabidopsis thaliana*, which include highly conserved amino acid sequences and functional domain structures (Ma et al., 2009; Park et al., 2009; Santiago et al., 2012). Several PYR/PYLs have been reported to be closely related to multiple ABA-mediated biological processes in *A. thaliana*, including AtPYR1, AtPYL1, AtPYL2, AtPYL3, AtPYL4, AtPYL8, and AtPYL13 (Park et al., 2009; Gonzalez-Guzman et al., 2012; Pizzio et al., 2013; Fuchs et al., 2014; Li et al., 2018). Moreover, PYLs members have been identified in *Zea mays* (13), *Oryza sativa* (13), and *Glycine max* (21) (He et al., 2018; Yadav et al., 2020; Zhang et al., 2020). Multiple ZmPYLs were strongly correlated with drought resistance in maize via participating in the ABA signaling pathway (He et al., 2018). As another core factor, PP2Cs are Mg^2+^-and Mn^2+^-dependent monomer enzymes, which are serine/threonine phosphatases that belong to the Mn^2+^/Mg^2+^-dependent PPM family (Ma et al., 2009; Shi, 2009). PP2Cs genes are co-expressed and are involved in lipid homeostasis (Liu et al., 2019). All PP2C-type phosphatases present 11 characteristic subdomains in the catalytic portion of the protein, where the catalytic domain is located at either the C-terminus or N-terminus in eukaryotes (Schweighofer et al., 2004; Xue et al., 2008). A total of 78 PPC2s members have been identified in *A. thaliana*, which have been clustered as 10 subfamilies (A–J). Subgroup A genes are associated with stress tolerance, particularly ABI1 and ABI2, which are strongly related with ABA signal transduction (Koornneef et al., 1984; Allen et al., 1999; Sun et al., 2011). The proteins encoded by *HAB1*, *HAB2*, and *AHG1* from group A have also been demonstrated to negatively regulate sucrose non-fermenting 1-related protein kinase 2 (SnRK2) kinases in response to ABA signaling (Saez et al., 2004, 2008; Umezawa et al., 2009). The AP2C1 from subgroup B can interact with MPK4 or MPK6 in response to wounding and pathogen stresses (Schweighofer et al., 2007). Subgroup C includes POL-type phosphatase, which is involved in flower development and the maintenance of stem cell polarity (Song et al., 2008; Gagne and Clark, 2010). Group D members constitute positive regulators in the ABA-mediated signaling pathways (Xue et al., 2008). SnRK2 is a unique plant protein kinase that belongs to the SNF1/AMPK family, which is involved in phosphorylation activities and the ABA signaling pathway that responds to various osmotic stresses (Kulik et al., 2011; Maszkowska et al., 2021). SnRK2 contains highly conserved N-termini kinase domains, an ATP-binding domain (GXGXXGX), and a serine/threonine activation loop. The C-terminus contains regulatory domains that are rich in acidic amino acids (AAs) (Liu et al., 2017; Mao et al., 2020; Wu et al., 2020). There are 10 SnRK2s members in *A. thaliana* (*AtSnRK2.1*–*2.10*), whereas *O. sativa*, *Z. may*, and *G. max* contain ten (*OsSAPK1*–*10*), eleven (*ZmSnRK2.1*–*2.11*), and twenty two (*GmSnRK2.1*–*2.22*), respectively (Zhao et al., 2017). Phylogenetic analysis revealed that the SnRK2 family includes three subgroups (subgroups I, II, and III), where subgroup III was strongly induced by ABA (Li et al., 2018; Maszkowska et al., 2021).

The PYR/PYL/RCAR family proteins can combine ABA to inhibit the phosphatase activity of PP2C, which negatively regulates ABA signaling primarily through phosphorylation and dephosphorylation (Miyazono et al., 2009). In the absence of ABA, PYR/PYL/RCAR does not combine with PP2Cs, which are released and bind with SnRK2s; thus, dephosphorylated and inactivated SnRK2s cannot phosphorylate downstream transcription factors. In the presence of ABA, ABA-bound PYR/PYL/RCAR combines with PP2Cs and inhibits its activity. The SnRK2s are released and phosphorylated the ABA response element-binding factor (ABFs) in response to ABA signaling (Miyazono et al., 2009; Park et al., 2009; Soon et al., 2012). A series of studies have revealed that PYR/PYL/RCAR members can interact with PP2Cs to inhibit the protein phosphatase activity, serving as a negative regulatory factor of PP2C (Ma et al., 2009; Park et al., 2009; Santiago et al., 2009; Nishimura et al., 2010). Furthermore, multiple PP2Cs have been observed to directly interact with groups II and III of SnRK2s in various combinations (Umezawa et al., 2009; Mizoguchi et al., 2010). Thus, these earlier studies show that the core regulators of ABA signaling physically connect at the gene family level, where PYR/PYL/RCAR negatively regulates PP2C, and PP2C negatively regulates SnRK2 (Umezawa et al., 2010).

The identification of PYL-PP2C-SnRK2s genes has been completed for various plant species, which has contributed to a better understanding of the roles of core components in the ABA signaling pathway. However, little is known about this gene family in Orchidaceae. In this study, we initially identified all PYL-PP2C-SnRK2s genes based on the genome database of *B. striata*. Bioinformatics analyses of genes and proteins were performed, including the physicochemical properties of proteins, gene structures, protein motif, conserved domain, phylogenetic relationships, chromosomal distribution, as well as intraspecific and interspecific collinearity. The expression patterns of various tissues, different germination stages, and responses to various abiotic stresses were analyzed. Several group A genes of BsPP2Cs, multiple BsPYLs, and BsSnRK2s were cloned, and subcellular localization was completed. Subsequently, yeast two-hybrid (Y2H) and Bimolecular fluorescence complementation (BiFC) assays were performed to analyze the protein interactions between BsPYLs and BsPP2Cs, as well as BsPP2Cs and BsSnRK2s. This study illustrates the role of ABA signaling in regulating germination and dormancy, as well as stress responses in *B. striata*.

## Materials and methods

### Identification and sequence analysis

Corresponding coding sequences (CDS) and amino acid sequences of AtPYL-PP2C-SnRK2s, OsPYL-PP2C-SnRK2s, and ZmPYL-PP2C-SnRK2s gene family were obtained from the TAIR database^1^ and the JGI Phytozome website,^2^ respectively. The *A. thaliana* sequences were employed as a query to search against the *B. striata* genome assembly of our lab via the BLASTn algorithm. The Hidden Markov Model (HMM) of PYL (PF10604), PP2C (PF00481), and SnRK2 (PF00069) from the Pfam database^3^ were applied as a query to blast all PYL-PP2C-SnRK2s containing sequences against the *B. striata* genome.

Further, the *e*-value cut-off of 0.01 was applied for homolog recognition, and only those that had complete PYL, PP2C, and SnRK2 domains were analyzed using InterProScan.^4^ The gene lengths, CDS, and amino acid sequences were counted. The gene structure of BsPYLs, BsPP2Cs, and BsSnRK2s were analyzed and displayed using TBtools v 0.58 with the gene sequences. The BsPYL-PP2C-SnRK2s protein sequences were employed to predict the conserved motifs using the online MEME^5^ program, the site distribution selected Zero or One Occurrence Per Sequence (zoops) and the number of motifs selected eight, with the expected *e*-values of less than 2 × 10^–30^, which was displayed with TBtools v 0.58. The conserved domain analysis was completed by NCBI-CDD search^6^ and displayed using TBtools.

### Multiple alignments and phylogenetic tree analysis

For multiple alignment analysis, SMART^7^ was used to acquire the core sequences of the PYL, PP2C, and SnRK2 domains, and the core sequences of the BsPYL-PP2C-SnRK2s were further analyzed via Geneious v9.1.4 and ClustalX v2.1 software programs. The Weblogo online program^8^ was used to display the characteristics of the domains. Three maximum likelihood (ML) phylogenetic trees of PYLs, PP2Cs, and SnRK2s were constructed using IQ-tree software (with 1,000 bootstraps) for statistical reliability to compare the evolutionary relationships, respectively. The phylogenetic trees of PYLs and SnRK2s involved AtPYLs, OsPYLs, ZmPYLs, and predicted BsPYLs, as well as AtSnRK2s, OsSnRK2s, ZmSnRK2s, and predicted BsSnRK2s protein. The phylogenetic tree of PP2Cs involved AtPP2Cs, OsPP2Cs and predicted BsPP2Cs. The ML phylogenetic trees of BsPYLs, BsPP2Cs, and BsSnRK2s protein sequences were also constructed to test the reliability of the results.

### Physicochemical properties of protein, *cis*-elements and Ka/Ks analysis

To analyze the promoter regions of the BsPYL-PP2C-SnRK2s, the upstream 2000 bp sequences of gene start codons were obtained from the whole-genome sequences of *B. striata*, and submitted to the PlantCARE database^9^ to identify the putative *cis*-element regulatory DNA elements in the promoter regions. The theoretical isoelectric point (PI) and molecular weights (MW) of the BsPYL-PP2C-SnRK2s proteins were predicted by the Compute PI/MW tool on the ExPASy server,^10^ resolution selected Average. The signal peptide prediction was performed by SignalP-5.0,^11^ organism group selected Eukarya. The transmembrane domain was predicted used TMHMM-2.0.^12^ The hydrophobicity/hydrophilicity prediction were completed by Hydropathy/Kyte and Doolittle tool on the ExPASy server.^13^ The protein secondary and tertiary structure prediction was performed used SOPMA^14^ and SWISS-MODEL,^15^ respectively. Furthermore, in order to analyze positive selection in the evolution of the BsPYL-PP2C-SnRK2s genes, the synonymous substitution rate (Ks) and non-synonymous substitution rate (Ka) values of homologous gene pairs were calculated with their amino acid sequences. After completing multiple sequence alignment using the online sites of Clustal Omega,^16^ PAL2NAL^17^ was used to calculate the Ka/Ks value, “Remove gaps, inframe stop codons” selected “Yes,” and “Calculate *d*_S_ and *d*_N_” also selected “Yes.”

### Chromosomal location and collinearity analysis

According to the *B. striata* genome assembly data, the chromosome mapping and the intraspecific collinearity analysis of BsPYLs, BsPP2Cs, and BsSnRK2s gene families was firstly performed by using TBtools software. Subsequently, after obtaining the genome assembly data of *A. thaliana*, *Z. mays*, and *Vanilla fragrans* from the NCBI database, the interspecific collinearity analysis was also conducted between *B. striata* and *A. thaliana*, *B. striata* and *Z. mays*, as well as *B. striata* and *V. fragrans*. Paralogous and orthologous gene pairs were also analyzed based on collinearity and phylogeny.

### Plant material and treatment

Seeds of *B. striata* were collected from Shiquan County of Shaanxi Province, China (N 33°8′12″, E 108°10′26″), which were sprouted on base soil and MS solid medium. A portion of the seeds was sterilized using 70% ethanol for 1 min followed by 0.15% HgCl_2_ solution for 7 min, rinsed with sterile water five times, sowed on the MS solid medium, and then cultured at 29 ± 1°C under a 12 h:12 h light/dark regime in a light incubator. The remaining seeds were sown directly on the soil in a greenhouse under natural lighting (12 h light/12 h dark) and 60–80% humidity.

For expression profile analysis of different tissues, roots, pseudobulbs, leaves, and flowers were collected separately from the 2-year-old plants at the flowering stage, with three replicates. The seedlings were collected separately at 0 day (BS0), 5 days (BS1), 10 days (BS2), 20 days (BS3), 28 days (BS4), and 35 days (BS5) after imbibition (DAI), which were used for germination-related expression profile analysis. A portion of seedlings were selected grown for 90 DAI in a greenhouse to complete the different treatment related expression profile analysis. For the hormonal treatment, the seedlings were sprayed with 100 μM ABA and 10 mM salicylic acid (SA), respectively. For the cold stress treatment, the seedlings were cultured at 4°C. The wounding treatment involved an incision (1 cm) that was made along the center of a seedling leaf. The seedlings were sprayed with 100 μM Cu_2_SO_4_ and 100 μM AgNO_3_ solutions for the heavy metal treatment. All the samples were collected after 0, 1, 3, 6, and 12 h under the different treatments with three replicates, respectively.

### RNA extraction and qRT-PCR analysis

The total RNA of all samples was extracted using the Polysaccharide and Polyphenols Plant Quick RNA Isolation Kit (TaKaRa, Dalian, China). After detecting the quality of RNA using 1% (p/v) gel electrophoresis, the concentration and absorbance ratios of the RNA (at A260/A280 and A260/A230) were measured using a NanoDrop 2000c spectrophotometer (Thermo Fisher Scientific, Waltham, MA, United States), where only high-quality RNA was used as a template for cDNA synthesis. A total of 1 μg RNA was reverse transcribed in a 20 μL volume using a PrimeScript™ RT Reagent Kit (TaKaRa, Dalian, China). The qRT-PCR primers were designed according to the CDS sequences using an online tool,^18^ which were showed in Supplementary Table 1. The qRT-PCR was completed via the Roche LightCycler 96 system (Roche, Basel, Switzerland) using the ChamQ™ SYBR^®^ qPCR Master Mix (Vazyme, Nanjing, China).

The cDNA was diluted 40 times to serve as a template, and the reaction system contained a total volume of 20 μL, which included 10 μL MasterMix, 0.5 μL forward primers, and 0.5 μL reverse primers (10 mM), 5.0 μL cDNA template, and 4.0 μL of sterile deionized water. The reaction conditions included pre-denaturation at 95°C for 30 s; 95°C for 5 s, followed by 60°C for 30 s, for a total of 45 cycles, and a final melting curve analysis. Each reaction proceeded with three technical replicates, and all relative gene expression values were calculated using the Equation 2^–Δ^ ^Δ^ *^Ct^* with *BsGADPH* as the internal reference gene.

### Subcellular localization analysis

Based on the *B. striata* genome database, the whole-length CDS sequences of *BsPYL2*, *BsPYL8*, *BsPP2C22*, *BsPP2C38*, *BsPP2C64*, *BsSnRK2.2*, and *BsSnRK2.4* were initially cloned into the TOPO vector, respectively. Subsequently, these genes were constructed to a pDONR207 vector (without termination codon) using a BP recombination reaction of the Gateway technology (Invitrogen, Carlsbad, United States). Finally, they were cloned into the pEarleyGate103 vector to form pEarleyGate103-*BsPYL2*, pEarleyGat e103-*BsPYL8*, pEarleyGate103-*BsPP2C22*, pEarleyGate103-*BsPP2C38*, pEarleyGate103-*BsPP2C64*, pEarleyGate103-*BsSnRK2.2*, and pEarleyGate103-*BsSnRK2.4* using the LR recombination reaction.

These vectors were employed as subcellular localization expressions, whereas pEarleyGate103 was used as a positive control. After embedding in a 1.0 μm gold powder suspension, the vectors were bombarded with PDS-1000 (Bio-Rad, Hercules, CA, United States) under a helium pressure of 1,100 psi into onion epidermal cells. The onion epidermal cells without any plasmid transformation were used as a negative control. The fluorescence of the green fluorescent protein (GFP) was observed under a Leica DM6000B microscope (Leica, Solms, Germany) at ∼475 nm wavelength following incubation at 28°C for 12–16 h in the dark. The primers used for gene cloning and vector construction were showed in Supplementary Tables 2,3.

### Protein interaction analysis

#### Yeast transactivation activity assay

Based on the LR recombination reaction, the pGBKT7-BsPP2Cs vectors were constructed based on pDONR207-BsPP2Cs (involving the termination codon). The primers were showed in Supplementary Table 4. After transforming into yeast strain AH109, the transcriptional activation test was conducted (as previously described) to identify the protein activation domain (AD). The colonies were cultured on SD/-Trp medium and screened on SD/-Trp/-His/-Ade medium supplemented with X-α-gal to evaluate the transcription activation, with the further selection of empty pGBKT7 vectors serving as negative controls.

#### Yeast two-hybrid assay

The recombinant vectors, pGADT7-BsPYLs and pGADT7-BsSnRK2s were constructed based on pDONR207-BsPYLs and pDONR207-BsSnRK2s (involving the termination codon) using the Gateway system. The primers were showed in Supplementary Table 4. Following co-transformation into yeast strain AH109 with pGBKT7-BsPP2Cs, the yeast cells were cultured on a double selection medium (SD/-Leu/-Trp) at 29°C for 3 days. The positive clones were then sequentially selected on SD/-Ade/-His/-Leu/-Trp medium with X-α-gal to test the interactions between BsPP2Cs and BsSnRK2s (BsPYLs), and 40 mM of 3-AT (3-amino-1,2,4-triazole) was added to the medium to inhibit transcriptional activation. The combination of pGBKT7-p53 and pGADT7 vectors served as positive controls, while the combination of pGBKT7-lam and pGADT7 served as negative controls.

#### Bimolecular fluorescence complementation assay

The pEarleyGate202-YC-BsPP2Cs and pEarleyGate201-YN-BsPYLs (BsSnRK2s) vectors were constructed based on pDONR207-BsPP2Cs and pDONR207-BsPYLs (BsSnRK2s) without a stop codon. The primers were showed in Supplementary Table 5. Equal concentrations of the YN-BsPYLs (YN-BsSnRK2s) and YC-BsPP2Cs recombinant plasmids were mixed prior to co-transformation, after which the mixed plasmids were bombarded into onion epidermal cells, and fluorescence signals were detected to search for interacting proteins according to method of subcellular localization analysis. The combination of YC-*BsPP2C22* (*38*)/YN, YN-BsPYLs/YC and YN-BsSnRK2s/YC vectors served separately as negative controls.

## Results

### Identification, nomenclature, and sequence features of BsPYL-PP2C-SnRK2s

A total of nine BsPYLs, 66 BsPP2Cs, and seven BsSnRK2s presumed genes were obtained from *B. striata* genome database. The number of BsPYL-PP2C-SnRK2s family members was lower than *A. thaliana* (14, 80, and 10) and *O. sativa* (12, 81, and 10). All BsPYLs, BsPP2Cs, and BsSnRK2s proteins were confirmed to contain a single domain by InterProScan, respectively. These genes were designated as *BsPYL1*∼*BsPYL9*, *BsPP2C1*∼*BsPP2C66*, and *BsSnRK2.1*∼*BsSnRK2.7*, respectively. The basic features of amino acid and gene sequences were identified, including the gene length, CDS, amino acid sequences, molecular weight (MW), and isoelectric point (pI). The amino acid sequence lengths of the *BsPYLs* gene ranged from 96 aa (*BsPYL5*) to 284 aa (*BsPYL4*), while *BsPP2Cs* and *BsSnRK2s* ranged from 124 aa (*BsPP2C49*) to 926 aa (*BsPP2C16*) and 335 aa (*BsSnRK2.4*) to 388 aa (*BsSnRK2.1*), respectively. The predicted molecular weight (MW) of the proteins ranged from 10.12 kDa (*BsPYL5*) to 30.95 kDa (*BsPYL4*), 13.92 kDa (*BsPP2C49*) to 102.94 kDa (*BsPP2C16*), and 37.81 kDa (*BsSnRK2.4*) to 44.63 kDa (*BsSnRK2.1*). The isoelectric points (p*I*) were predicted to range from 5.15 (*BsPYL2*) to 9.24 (*BsPYL5*), 4.48 (*BsPP2C25*) to 9.97 (*BsPP2C49*), and 4.89 (*BsSnRK2.5*) to 9.28 (*BsSnRK2.7*) (Supplementary Table 6).

### Phylogenetic analysis of BsPYL-PP2C-SnRK2s

**FIGURE 1:**
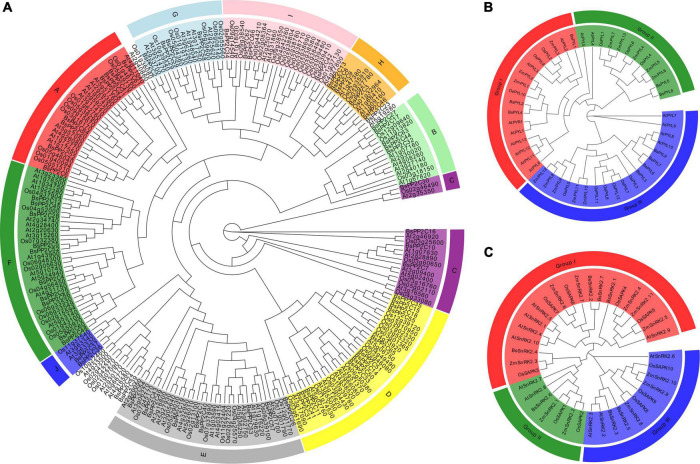
Phylogenetic trees of PP2Cs, PYLs, and SnRK2s gene families. **(A)** The maximum likelihood tree of PP2Cs include 78 members from *A. thaliana* (At), 80 members from *O. sativa* (Os), and 66 members from *B. striata* (Bs). The maximum likelihood tree of PYLs **(B)** and SnRK2s **(C)** gene families in *A. thaliana* (At), *O. sativa* (Os), *Z. mays* (Zm), and *B. striata* (Bs). The ML phylogenetic trees were constructed using IQ-tree software with 1000 replicates. Different leaf background colors indicate the different subgroups.

To determine and elucidate the evolutionary relationship of BsPYL-PP2C-SnRK2s, all identified amino acid sequences of 48 PYLs, 227 PP2Cs, and 38 SnRK2s from *B. striata*, *A. thaliana*, *O. sativa*, and *Z. mays* were selected to construct three ML phylogenetic trees with 1000 bootstraps (Figure 1). According to the branching characteristics, a total of 227 PP2Cs were clustered into 10 subgroups (A–J) (Figure 1A). The largest subgroup (F) included 33 members (10 BsPP2Cs, 11 OsPP2Cs, and 12 AtPP2Cs). Subgroup A included eight BsPP2Cs, nine OsPP2Cs, and nine AtPP2Cs, while subgroup B included five BsPP2Cs, three OsPP2Cs, and six AtPP2Cs. A total of 48 PYLs were clustered into three subgroups (I–III) (Figure 1B), which included 13 (subgroup I), 16 (subgroup II), and 19 members (subgroup III), respectively. Subgroup III contained four BsPYLs, four AtPYLs, six OsPYLs, and five ZmPYLs. A total of 38 SnRK2s were also clustered into three subgroups (I–III) (Figure 1C), which included 20 (subgroup I), seven (subgroup II), and 11 members (subgroup III), respectively. Furthermore, BsPYL1 was close to AtPYL2 and AtPYL3, which were in the same clade; BsPP2C6 and BsPP2C22 were close to AtABI1 (AT4g26080) and AtABI2 (AT5g57050) in the phylogenetic clade, while BsPP2C20 and BsPP2C64 were phylogenetically close to AtAHG3 (AT3g11410), which played crucial roles in the seed germination of *A. thaliana*.

### Gene structure, conserved domain, motif composition, multiple sequence alignments, *cis*-acting elements, and Ka/Ks analysis of BsPYL-PP2C-SnRK2s

**FIGURE 2:**
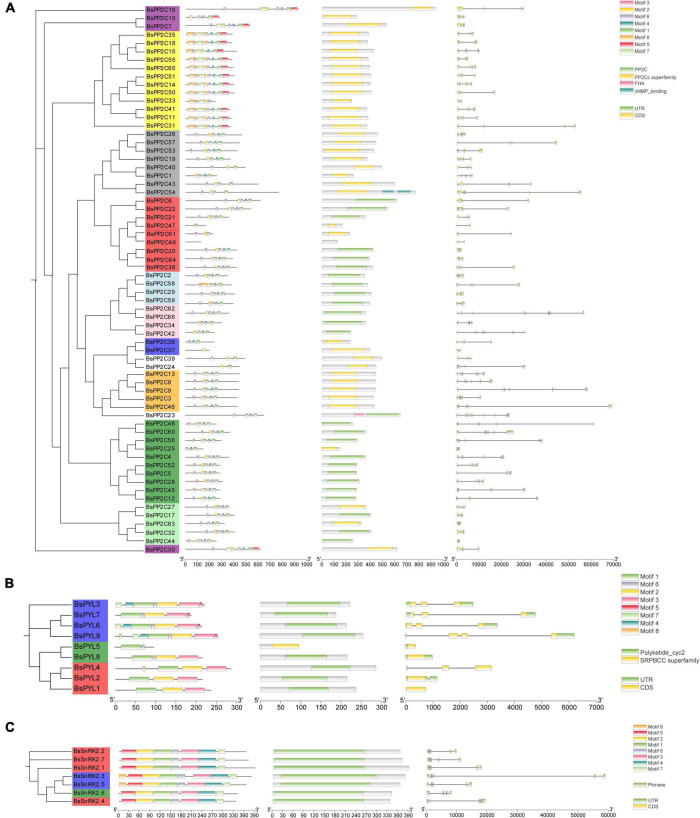
Phylogenetic trees, motifs, conserved domain and gene structures of BsPP2Cs **(A)**, BsPYLs **(B)**, and BsSnRK2s **(C)** gene families. For the phylogenetic trees, different leaf background colors indicate various subgroups. For the motifs, different colored boxes indicate various conserved motifs. For the conserved domain, colored boxes indicate different conserved domain. For the gene structure, green boxes, gray lines, and yellow boxes represent exons, introns, and UTRs, respectively. The results were displayed using TB tools v 0.58.

To better understand the BsPYL-PP2C-SnRK2s gene families, the gene structures, motifs and conserved domain were analyzed. Three ML phylogenetic trees with the BsPYL, BsPP2C, and BsSnRK2 protein sequences were constructed, respectively (Figure 2). According to the results, genes of the same subfamily contain the similar distribution of conserved domains, the *BsPYL1*, *BsPYL5*, and *BsPYL8* contained no intron; *BsPYL3*, *BsPYL4*, *BsPYL6*, and *BsPYL7* had two introns; and *BsPYL2* and *BsPYL9* possessed one and three introns, respectively, whereas *BsPYL1* and *BsPYL4* had no UTR regions. All members of the BsPP2Cs family contained introns (Figure 2).

Secondary structure elements analysis of BsPYL-PP2C-SnRK2s protein showed that alpha helix and random coil were the main components of the secondary structure of BsPYL-PP2C-SnRK2s proteins (Supplementary Table 7). The signal peptide prediction showed that BsPYL-PP2C-SnRK2s proteins had no signal peptide structure, except for BsPP2C37 (Supplementary Figure 2). Furthermore, there were nine BsPP2Cs, and one BsPYL had transmembrane domain, suggested that most proteins of the BsPYL-PP2C-SnRK2s were non-transmembrane proteins (Supplementary Figure 3). The hydrophobicity/hydrophilicity prediction of the BsPYL-PP2C-SnRK2s proteins suggested that all proteins showed no obvious hydrophilic and hydrophobic characteristics (Supplementary Figure 4). The tertiary structure prediction exhibited that the same subfamily members contained similar tertiary structures (Supplementary Figure 5).

The *cis*-acting elements of the promoter region (upstream 2000 bp sequences of the start codon) were analyzed (Supplementary Figure 6). The results indicated that BsPP2Cs, BsPYLs, and BsSnRK2s genes could respond to a variety of abiotic stresses. In particular, a total of 46 (69.70%) BsPP2Cs, eight (88.89%) BsPYLs, and all BsSnRK2s genes contained ABRE elements to respond to ABA signaling; a total of 24 (36.36%), four (44.44%) BsPYLs, and three (42.86%) BsSnRK2s genes contained TCA-elements to respond to SA signaling. A total of 41 (62.12%) BsPP2Cs, three (33.33%) BsPYLs, and three (42.86%) BsSnRK2s genes contained the WUN-motif to respond to wound damage; a total of 24 (51.52%) BsPP2Cs, five (55.56%) BsPYLs, and three (42.86%) BsSnRK2s genes contained LTR, which were involved in low-temperature responsiveness.

### Chromosomal localization and collinearity analysis of BsPYL-PP2C-SnRK2s

**FIGURE 3:**
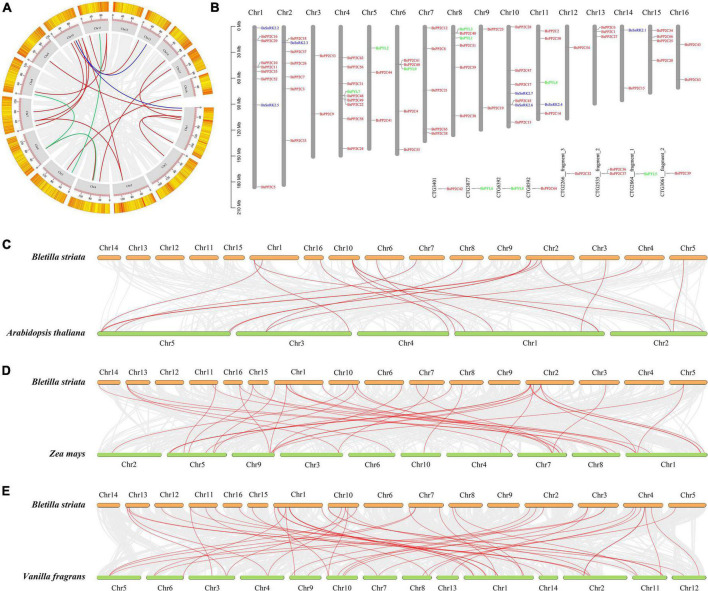
Chromosomal localization and collinearity analysis of BsPP2Cs, BsPYLs, and BsSnRK2s gene families. For intraspecific collinearity analysis **(A)** and chromosomal localization **(B)**, gray boxes (gray columns in panel **B**) represent the 16 pseudochromosomes of *B. striata*, and red, green, and blue lines represent collinear relationships and localization in BsPP2Cs, BsPYLs, and BsSnRK2s gene families, respectively. Interspecific collinearity analysis of BsPP2Cs, BsPYLs, and BsSnRK2s between *B. striata* and *A. thaliana* **(C)**, *Z. mays* **(D)**, as well as *V. fragrans* **(E)**. Red lines represent collinear relationships of orthologous gene pairs.

According to the *B. striata* genome assembly and annotation information, the BsPYL-PP2C-SnRK2s gene family was localized on 16 pseudochromosomes of *B. striata* (chr1-chr16) (Figure 3B). There were six BsPYLs, 60 BsPP2Cs, and seven BsSnRK2s precisely located on pseudochromosomes. Six BsPYLs were located on chr4-5, chr8, and chr11, while seven BsSnRK2s were located on chr1-2, chr10-11, and chr14, and 60 BsPP2Cs were all located on pseudochromosomes. For the BsPYL gene family, chr8 contained two genes, whereas other chromosomes contained one gene; chr1 and chr3 contained two *BsSnRK2* genes, while chr2, chr11, and chr14 contained one BsSnRK2 gene. The chr1 and chr4 had the greatest BsPYL-PP2C-SnRK2s gene localization (nine genes), where seven BsPP2Cs and two BsSnRK2s were located on chr1, *BsPYL7* and eight BsPP2Cs were located on chr4, while only chr12 had a single gene of the BsPP2C family (*BsPP2C54*). The chr3, chr7, chr9, chr12, chr13, chr15, and chr16 had only BsPP2Cs family localization, while only chr11 contained BsPYLs, BsPP2Cs, and BsSnRK2s at the same time.

The results of intraspecific collinearity analysis revealed that BsPYL, BsPP2C, and BsSnRK2 families had three, 14, and two pairs paralogous genes, respectively (Figure 3A). Sequentially, interspecific collinearity analysis was performed to elucidate the evolutionary relationship between *B. striata* and other plants (Figures 3C–E). The results indicated that there were one, 15, and three pairs of orthologous genes of the PYL, PP2C, and SnRK2 families between *B. striata* and *A. thaliana* (Figure 3C). There were 31 and seven pairs orthologous of PP2C and SnRK2 genes between *B. striata* and *Z. mays* (Figure 3D), while four, 41, and three pairs orthologous genes of the PYL, PP2C, and SnRK2 families between *B. striata* and *V. fragrans* (Figure 3E).

### Tissue-specificity expression of BsPYL-PP2C-SnRK2s

**FIGURE 4:**
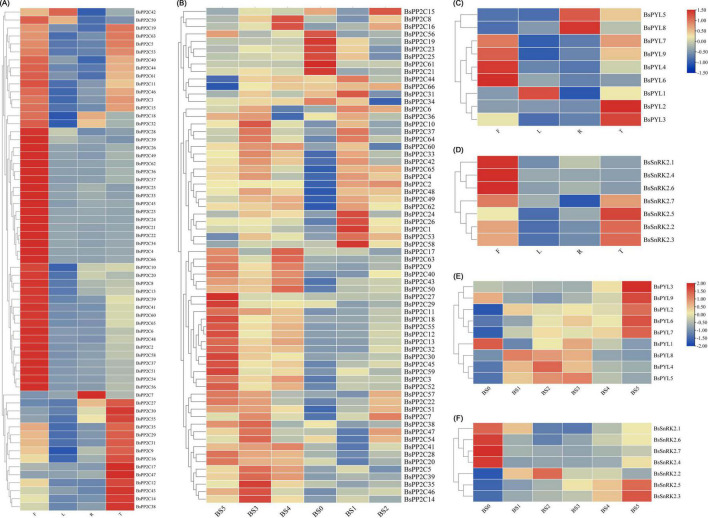
Expression profile heatmap with hierarchal clustering of BsPP2Cs **(A,B)**, BsPYLs **(C,E)**, and BsSnRK2s **(D,F)** in different tissues and different germination stages of *B. striata*. R, T, L, and F in panels **(A,C,D)** represented root, pseudobulb, leaf, and flower, while BS0∼BS5 represented different germination stages, respectively. The relative expression for each genes is depicted by color intensity in each field. Higher values are represented by red whereas lower values are represented by blue.

According the heatmap of qRT-PCR analysis (Figure 4), four BsPYLs (*BsPYL4*, *BsPYL6*, *BsPYL7*, and *BsPYL9*) were significantly higher in flowers, while *BsPYL2* and *BsPYL3* were highly expressed in pseudobulbs (Figure 4C). In the BsPP2Cs family, 14 BsPP2Cs genes had the highest expression levels in pseudobulbs, whereas *BsPP2C7* had the highest expression levels in roots. There were 33 BsPP2Cs genes with the highest expression levels in flowers, while 11 BsPP2Cs genes had higher expression levels in flowers and pseudobulbs. *BsPP2C42* and *BsPP2C50* were significantly higher in flowers and leaves, while *BsPP2C18* and *BsPP2C32* were significantly higher in flowers and roots (Figure 4A). For the BsSnRK2s family, *BsSnRK2.1*, *BsSnRK2.4*, and *BsSnRK2.6* had the highest expression levels in flowers, whereas *BsSnRK2.2*, *BsSnRK2.3*, *BsSnRK2.5*, and *BsSnRK2.7* exhibited higher expression levels in flowers and pseudobulbs (Figure 4D).

Furthermore, the analysis of BsPYL-PP2C-SnRK2s expression patterns during seed germination was conducted. There were five BsPYLs (*BsPYL2*, *BsPYL3*, *BsPYL6*, *BsPYL7*, and *BsPYL9*) that were significantly higher in BS5. The expressions of *BsPYL4*, *BsPYL5*, and *BsPYL8* were significantly higher in BS1∼BS3, while *BsPYL1* was highly expressed for BS0 and BS3 (Figure 4E). For the BsPP2Cs and BsSnRK2s families, *BsSnRK2.3* and *BsSnRK2.5* were highly expressed in BS4 and BS5, while the expression of *BsSnRK2.2* was significantly higher in BS1 and BS2 (Figure 4F). There were 18 BsPP2Cs genes, including *BsPP2C6*, *BsPP2C44*, and *BsPP2C64* that exhibited higher expression levels in BS1 and BS3. A total of 13 BsPP2Cs genes including *BsPP2C27* had the highest expression levels in BS5, while 15 BsPP2Cs genes including *BsPP2C20*, *BsPP2C22*, and *BsPP2C38* were more highly expressed in BS3, which belonged to group A of BsPP2Cs (Figure 4B).

### BsPYL-PP2C-SnRK2s response to multiple different stress treatments

**FIGURE 5:**
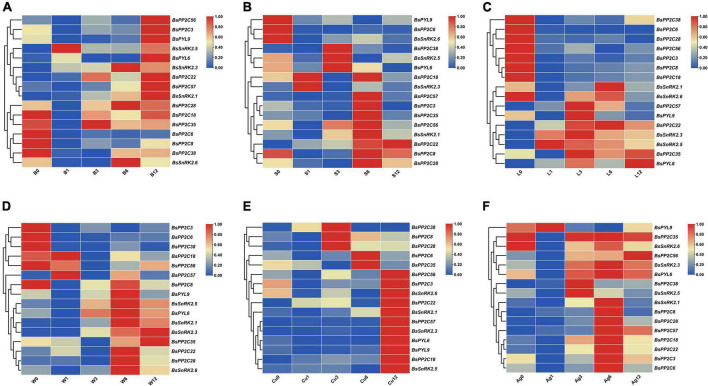
Expression profile heatmap with hierarchal clustering of selected two BsPYLs, ten BsPP2Cs, and four BsSnRK2s genes in response to different stress treatments for 0, 1, 3, 6, and 12 h, including ABA (**A**, B0–B12), SA (**B**, S0–S12), low temperature (**C**, L0–L12), wounding (**D**, W0–W12), CuSO_4_ (**E**, Cu0–Cu12), and AgNO_3_ (**F**, Ag0–Ag12). The relative expression for each genes is depicted by color intensity in each field. Higher values are represented by red whereas lower values are represented by blue.

To evaluate the expression pattern responses to different hormone (ABA and SA) and abiotic stress treatments (low temperature, wounding, CuSO_4_, and AgNO_3_), two BsPYLs, 10 BsPP2Cs, and four BsSnRK2s genes were randomly selected based on their phylogenetic distance and qRT-PCR was performed (Figure 5; Supplementary Figure 7). According to the results, these genes had different expression levels under various treatments. For the BsPP2C family, the *BsPP2C6*, *BsPP2C22*, and *BsPP2C38* significantly responded to all treatments, while the responses of *BsPP2C18* and *BsPP2C35* were not obvious under partial treatments. For the BsSnRK2 family, the responses of *BsSnRK2.1*, *BsSnRK2.3*, *BsSnRK2.5*, and *BsSnRK2.6* were significant under various treatments except for *BsSnRK2.3*, which had no response under the SA treatment. For the BsPYLs family, *BsPYL6* and *BsPYL9* responded significantly to various treatments, expect for *BsPYL9*, which did not respond to the low temperature and AgNO_3_ treatments. Moreover, under the ABA treatment, the expression level of these genes was significantly higher at 12 h compared with the other treatment times. The *BsPP2C6* and *BsPP2C38* had similar expression patterns under the various treatments and responded to them all, while *BsPP2C6* and *BsPP2C22* exhibited an opposite expression profile. *BsPP2C6* and *BsPP2C38* were significantly downregulated by ABA, low temperature and wounding, while *BsPP2C22* was significantly upregulated under the various treatments. These results revealed that subgroup A of BsPP2Cs could respond to multiple abiotic stresses.

### Subcellular localization of BsPYL-PP2C-SnRK2s family

**FIGURE 6:**
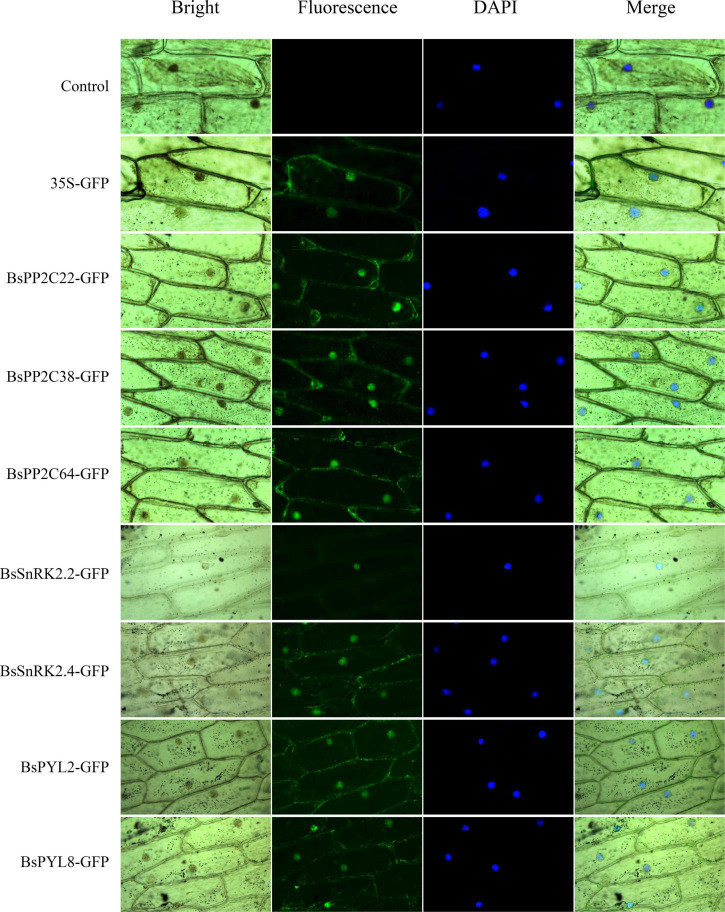
Subcellular localization of BsPP2C22, BsPP2C38, BsPP2C64, BsSnRK2.2, BsSnRK2.4, BsPYL2, and BsPYL8 in onion epidermal cells. Green fluorescence was observed at ∼475 nm, and DAPI was observed at ∼350 nm.

To further understand protein function, three BsPP2C genes (*BsPP2C22*, *BsPP2C38*, and *BsPP2C64*), two BsPYL genes (*BsPYL2* and *BsPYL8*), and two BsSnRK2 genes (*BsSnRK2.2* and *BsSnRK2.4*) were cloned according to the primers in Supplementary Table 2. The BsPP2Cs-GFP, BsPYLs-GFP, and BsSnRK2s-GFP expression vectors were constructed according to the primers in Supplementary Table 3. These vectors were transformed into onion epidermis for transient expression. The results of subcellular localization showed that the green fluorescence of the positive control was localized in the nucleus and membrane, while no green fluorescence was observed in the negative control group. Compared with the control group, only *BsSnRK2.2*-GFP was concentrated in the nucleus, while BsPP2C22-GFP, BsPP2C38-GFP, BsPP2C64-GFP, BsPYL2-GFP, BsPYL8-GFP, and BsSnRK2.4-GFP were localized in both the nucleus and membrane (Figure 6).

### BsPP2C22 and BsPP2C38 interact with multiple BsPYL and BsSnRK2 proteins

**FIGURE 7:**
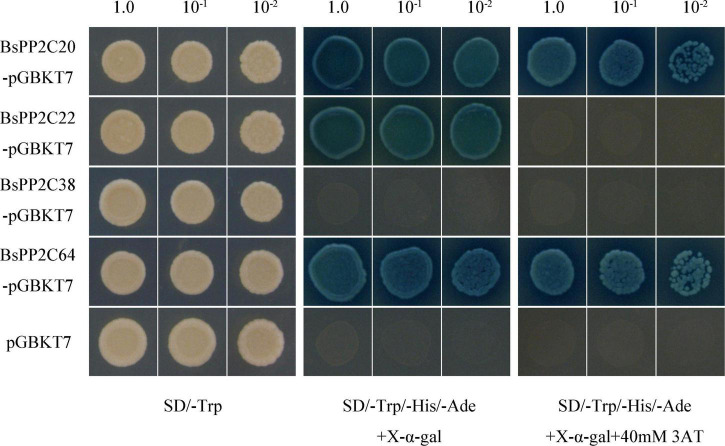
Yeast Transactivation Activity Assay of BsPP2C20, BsPP2C22, BsPP2C38, and BsPP2C64. The BsPP2C20, BsPP2C22, BsPP2C38, and BsPP2C64 were fused with the DNA-binding domain (BD). BD-BsPP2C20 + AD, BD-BsPP2C22 + AD, BD-BsPP2C38 + AD, and BD-BsPP2C64 + AD, respectively, were grown on SD/-Trp media, SD/-Trp/-Ade/-His + X-α-gal, and SD/-Trp/-Ade/-His + X-α-gal + 40 μM 3-AT media.

Firstly, the BsPP2C20-BD, BsPP2C22-BD, BsPP2C38-BD, and BsPP2C64-BD vectors were constructed (Supplementary Table 3), and a transactivation activity assay was performed in yeast. According to the results, the yeast cells transformed with pGBKT7-BsPP2C20, pGBKT7-BsPP2C22, and pGBKT7-BsPP2C64 grew normally and presented a blue color on the SD/-Trp/-His/-Ade medium supplemented with X-α-gal, while pGBKT7-BsPP2C38 grew only on the SD/-Trp medium. This demonstrated that BsPP2C20, BsPP2C22, and BsPP2C64 had transcriptional activation activity, whereas BsPP2C38 had none. When 40 mM of 3-AT was added to the SD/-Trp/-His/-Ade medium, the transcriptional activation activity of BsPP2C22 was inhibited, and did not grow normally (Figure 7).

**FIGURE 8:**
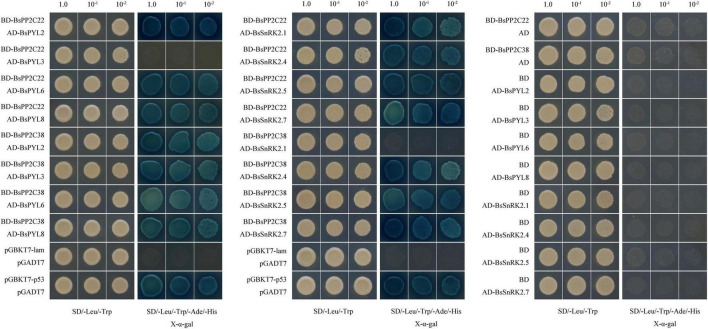
Yeast two-hybrid (Y2H) assays to test the interactions between BsPP2C22 (BsPP2C38) and BsPYLs, as well as BsPP2C22 (BsPP2C38) and BsSnRK2s. BsPP2C22 (BsPP2C38) was fused with the DNA-binding domain (BD), while BsPYLs and BsSnRK2s were fused with the activation domain (AD). Positive control (pGBKT7-p53 + pGADT7), negative control (pGBKT7-lam + pGADT7), BD-BsPP2C38 + AD-BsPYLs (BsSnRK2s) groups, BD-BsPP2C38 + AD group, and BD + AD-BsPYLs (BsSnRK2s) groups and were grown on SD/-Leu/-Trp/-Ade/-His + X-α-gal media. BD-BsPP2C22 + AD-BsPYLs (BsSnRK2s) groups and BD-BsPP2C22 + AD were grown on SD/-Leu/-Trp/-Ade/-His + X-α-gal + 40μM 3-AT media.

**FIGURE 9:**
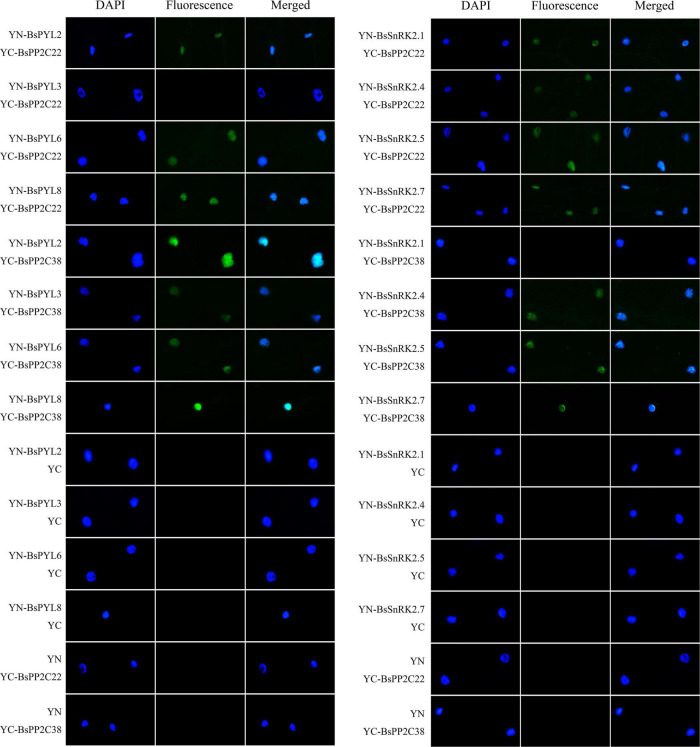
Bimolecular fluorescent complementation (BiFC) experiments in onion epidermal cells to test the interactions between BsPP2C22 (BsPP2C38) and BsPYLs, as well as between BsPP2C22 (BsPP2C38) and BsSnRK2s. BsPP2C22 (BsPP2C38) was fused with the C-terminal of fluorescein (YC). BsPYLs and BsSnRK2s were fused with the N-terminal of fluorescein (YN). Green fluorescence was observed at ∼475 nm, and DAPI was observed at ∼350 nm.

The BsPYL2, BsPYL3, BsPYL6, BsPYL8, BsSnRK2.1, BsSnRK2.4, BsSnRK2.5, and BsSnRK2.7 were cloned separately into pGADT7 (Supplementary Table 3), and the Y2H assay was performed to test the interactions between BsPP2C22 (BsPP2C38) and BsPYLs, as well as BsPP2C22 (BsPP2C38) and BsSnRK2s. The results showed that BsPP2C22 interacted with BsPYL2, BsPYL6, BsPYL8, BsSnRK2.1, BsSnRK2.4, BsSnRK2.5, and BsSnRK2.7, while did not with BsPYL3. BsPP2C38 interacted with BsPYL2, BsPYL3, BsPYL6, BsPYL8, BsSnRK2.4, BsSnRK2.5, and BsSnRK2.7, while did not interact with BsSnRK2.1 (Figure 8). Finally, the pEarleyGate202-YC-BsPP2C22 (BsPP2C38) vectors were constructed separately based on pDONR207-BsPP2C22 and pDONR207-BsPP2C38 without stop codons. The CDS of BsPYL2, BsPYL3, BsPYL6, BsPYL8, BsSnRK2.1, BsSnRK2.4, BsSnRK2.5, and BsSnRK2.7 without stop codons were recombined separately into the pEarleyGate201-YN (Supplementary Table 5). The BiFC assay in onion epidermal cells was performed to verify the Y2H results, which were consistent with those of the Y2H (Figure 9).

## Discussion

The PYL-PP2C-SnRK2s family exists in many species to participate in a variety of ABA-mediated biological processes. For this study, we identified and characterized PYL-PP2C-SnRK2s family based on the genome databases of *B. striata*. They can perform many different biological functions and the expression profiles varied for different tissues and seed germination stages of *B. striata*. The results of Y2H and BiFC assays suggested two important members of BsPP2Cs, BsPP2C22 and BsPP2C38 could interact with BsPYLs and BsSnRK2s in response to multiple abiotic stresses.

### PYL-PP2C-SnRK2s gene family was contracted in *Bletilla striata*

As one of the core regulation pathways of plants, ABA signaling pathway and its various regulatory mechanisms have been investigated for multiple model plants, including *A. thaliana*, *O. sativa*, and *Z. mays*. The PYL-PP2C-SnRK2s gene family has been documented as a core regulatory network of the ABA signaling pathway in many species; however, there are no reports known for Orchidaceae. Herein, for the first time, we identified and characterized the PYL-PP2C-SnRK2s gene family based on the whole genome sequence of the *B. striata* orchid. In this study, a total of nine PYLs, 66 PP2Cs, and seven SnRK2s were identified from the *B. striata* genome database, which was classified into three, ten, and three subfamilies, respectively. Compared with *A. thaliana* (14, 80, and 10), *O. sativa* (12, 78, and 10), and *Z. mays* (12, 97, and 10), *G. max* (21, 36, and 21), *Clonorchis sinensis* (14, 84, and 8), and *Musa nana* (24, 87, and 11), the number of PYL-PP2C-SnRK2s genes was smaller (Figure 1; Xue et al., 2008; Saha et al., 2014; Hu et al., 2017; Zhao et al., 2017; He et al., 2018; Dittrich et al., 2019; Fan et al., 2019; Xu et al., 2020; Zhang et al., 2020). In other words, the PYL-PP2C-SnRK2s gene family was contracted in *B. striata*. The gene structure analysis results revealed that all BsPYLs, BsPP2Cs, and BsSnRK2s genes contained introns, except for *BsPYL1*, *BsPYL5*, and *BsPYL8*. Most members within the same subgroup shared similar intron/exon structures, conserved domain and common motifs, and there were obvious differences in the motifs of different subfamilies. This strongly supported their close evolutionary relationships and the reliability of our subfamily phylogenetic classification.

Moreover, motif analysis indicated that the START-like, PPM-type phosphatase, and protein kinase domains were present in all BsPYLs, BsPP2Cs, and BsSnRK2s, respectively (Schweighofer et al., 2004; Radauer et al., 2008; Mao et al., 2020; Yadav et al., 2020). There were significant variations in gene length, although the CDS length had no obvious differences, in contrast to *A. thaliana*, *O. sativa*, *Z. Mays*, and *G. max*. The CDS length of BsPYLs, BsPP2Cs, and BsSnRK2s were around 700, 1000, and 1100 bp, respectively, while the gene length ranged from 362 (*BsPYL5*) to 6196 bp (*BsPYL9*), 1051 (*BsPP2C44*) to 68944 bp (*BsPP2C46*), and 8087 (*BsSnRK2.6*) to 58560 bp (*BsSnRK2.4*) (Supplementary Table 1; Figure 2). We speculated that this was because of the *B. striata* genome was more complex. According to the results of the phylogenetic analysis of three species (*B. striata*, *A. thaliana*, and *O. sativa*), a total of 227 PP2Cs genes were grouped into ten subfamilies (Figure 1). There were eight PP2C genes from *B. striata* were identified and classified into subfamily A, compared with *A. thaliana* (9) and *O. sativa* (10), it was also contracted in *B. striata*. This suggested that group A of BsPP2Cs may have more abundant functions in *B. striata*.

### PYL-PP2C-SnRK2s gene family has multiple biological functions in *Bletilla striata*

Over the last 20 years, the group A members of PP2Cs were demonstrated to regulate plant growth, dormancy, germination, and stress responses by participating in the ABA signaling pathway (Park et al., 2009; Bhaskara et al., 2012; Nishimura et al., 2018; He et al., 2019). AtABI1 (AT4g26080), AtABI2 (AT5g57050), and AtAHG3 (AT3g11410) were reported to play crucial roles in the germination of *A. thaliana* seeds, while *abi1-1* and *abi1-2* mutants had ABA-insensitive germination, and *ahg3* mutants showed a strong hypersensitive ABA phenotype during germination (Koornneef et al., 1984; Allen et al., 1999; Yoshida et al., 2006; Sun et al., 2011). These results indicated that BsPP2C6, BsPP2C20, BsPP2C22, BsPP2C38, and BsPP2C64 may also play important roles in the germination of *B. striata* seeds, as they were phylogenetically close to AtABI1, AtABI2, and AtAHG3, respectively. Additionally, previous studies revealed that the group B members of the PP2C in *A. thaliana* regulated seed germination [AtPP2C5 (At2g40180)] (Brock et al., 2010), stomata development [AtAP2C3 (At2g40180)] (Umbrasaite et al., 2010), defense responses, and innate immunity [AtAP2C1 (At2g30020)] (Schweighofer et al., 2007; Sidonskaya et al., 2016; Shubchynskyy et al., 2017) via the mitogen-activated protein kinase (MAPK) signaling pathway. Five BsPP2Cs, six AtPP2Cs, and three OsPP2Cs were clustered into subgroup B, which implied that these genes could have similar functions. Subgroup C contained seven AtPP2Cs, four BsPP2Cs, and six OsPP2Cs genes, which were reported to contribute to the development of stem cells, meristems, and organs (Song and Clark, 2005; Song et al., 2008, 2020). BsPP2C23 and AtKAPP (At5g19280) were clustered as orthologous genes, implying that they might perform similar functions, which participates in Na^+^ stress and defense responses (Manabe et al., 2007; Gramegna et al., 2016).

From the phylogenetic analysis of the PYL and SnRK2 family in four different species (*B. striata*, *A. thaliana*, *O. sativa*, and *Z. Mays*), all 48 PYLs genes and 38 SnRK2s genes were classified into three subgroups (group I∼group III). The data showed that groups I, II, and III of the PYL family contained 13, 16, and 19 genes, respectively, while groups I, II, and III of the SnRK2 family separately contained 20, seven, and 11 genes. BsPYL1, BsPYL2, and BsPYL4 were clustered into the same subfamily as AtPYR1, AtPYL1, AtPYL2, AtPYL3, and ZmPYL3, while BsPYL5 and BsPYL8 were clustered into the same subfamily as AtPYL4, AtPYL13, and ZmPYL13. BsPYL3, BsPYL6, BsPYL7, and BsPYL9 were clustered into the same subfamily as AtPYL8, ZmPYL8, ZmPYL9, ZmPYL10, and ZmPYL12. Among these genes, *AtPYL2* was the orthologous gene of *BsPYL1*, and *AtPYL8* was the orthologous gene of *BsPYL9*. It was confirmed that these PYLs genes from *A. thaliana* and *Z. Mays* were closely related to the ABA-mediated drought response and stomatal aperture (Park et al., 2009; Gonzalez-Guzman et al., 2012; Pizzio et al., 2013; He et al., 2018; Li et al., 2018). This proved that these PYLs genes from *B. striata* most likely had similar functions. Furthermore, five AtSnRK2s genes were reported to be activated by ABA signaling to participate in drought stress responses, particularly AtSnRK2.2, AtSnRK2.3, and AtSnRK2.6. Further, AtSnRK2.2 and AtSnRK2.3 regulated seed germination and seedling growth, AtSnRK2.6 participated stomatal regulation via the ABA signaling pathway in *A. thaliana*, and ABA responses were eliminated in a *snrk2.2/2.3/2.6* triple-mutant (Fujii and Zhu, 2009; Nakashima et al., 2009). Likewise, ZmSnRK2.1/2/8/10 were reported to be strongly activated by ABA, while ZmSnRK2.4/6/7/11 did not strongly respond to ABA (Wang et al., 2018). Therefore, we believed that BsSnRK2.3, BsSnRK2.5, and BsSnRK2.6 might have stronger responses to ABA than other BsSnRK2s, as BsSnRK2.3 and BsSnRK2.5 were clustered into the same subfamily with AtSnRK2.2, AtSnRK2.3, AtSnRK2.6, ZmSnRK2.8, and ZmSnRK2.10, whereas BsSnRK2.6 was clustered into the same subfamily as ZmSnRK2.1 and ZmSnRK2.2. According to phylogenetic trees and intraspecific collinearity analysis, the BsPYL, BsPP2C, and BsSnRK2 families contained three, 24, and two pairs of paralogous genes, respectively. These pairs had a Ka/Ks value < 1 (Figure 3; Supplementary Table 3), which suggested that these genes were purify selection (Goldman and Yang, 1994). Thus, these genes may have played an important role in the evolution of *B. striata*. Moreover, the results of interspecific collinearity analysis revealed that there were more orthologous gene pairs between *B. striata* and *V. fragrans*, in contrast to between *B. striata* and *A. thaliana*, or between *B. striata* and *Z. mays*, which also suggested closer genetic kinship between species.

### The expression profiles of BsPYL-PP2C-SnRK2s varied for different tissues and seed germination stages of *Bletilla striata*

From the qRT-PCR results, the expression patterns of BsPYL-PP2C-SnRK2s gene families in different *B. striata* tissues revealed significant differences, which implied that there were functional differences between different genes (Figure 4). For BsPYL, a similar tendency was shown in the same subgroup genes. Group II of BsPYL was higher expressed in roots, while group III exhibited higher expression level in flowers, and group I showed significant overexpression in pseudobulbs. Likewise, similar expression patterns were observed for the BsSnRK2 family, which indicated that genes from the same subgroup could perform similar functions for different biological processes. As a unique, and key tissue of many orchids, the pseudobulb has a significant role in water storage and contributes to the plant-water balance and tolerance against drought stress (Zhang et al., 2018). The results showed that two BsPYLs genes, 14 BsPP2Cs genes, and four BsSnRK2s genes exhibited higher expression levels in pseudobulbs compared with other tissues, including multiple different subgroups of the BsPYL, BsPP2C, and BsSnRK2 families. This indicated that these genes were strongly associated with the formation, development, and growth of *B. striata* pseudobulbs, as well as being closely related to water storage and drought stress.

The expression patterns of BsPYL-PP2C-SnRK2s gene families at different *B. striata* germination stages were also analyzed, as they comprise the core network of the ABA signaling pathway and are strongly associated with seed germination and seedling development (Figure 4). According to the results, several genes (BsPYL4, BsPYL5, BsPYL8, BsPP2C17, BsPP2C20, BsPP2C38, and BsSnRK2.2, etc.) exhibited higher expression level during the protocorm formation process (BS3), indicated that these genes were strongly related to formation and development of protocorm, which is a sign and a critical juncture in the normal germination of orchid seeds (Rasmussen and Rasmussen, 2009; Yeung, 2017). Furthermore, more than half the members of the BsPP2C family exhibited higher expression level in BS5, which suggested that the BsPP2C family play an important role for early seedling establishment of *B. striata*.

### BsPP2C22 and BsPP2C38 interact with BsPYLs and BsSnRK2s in response to multiple abiotic stresses

Additionally, we found that the BsPP2Cs, BsPYLs, and BsSnRK2s genes contained several *cis*-acting elements related to hormone responses and abiotic stresses, including ABA, GA (gibberellin), SA, MeJA, wounding, light, drought, and low temperatures, which implied that these genes could respond to multiple treatments and stresses (Supplementary Figure 6). As core components of the ABA signal pathway, most BsPP2Cs (69.70%) and BsPYLs (88.89%), as well as all BsSnRK2s genes contained ABRE elements to respond to ABA signaling. In particular, all group A of BsPP2Cs contained ABRE elements, which verified the rationality of our phylogenetic tree analysis, as earlier studies reported that group A of the PP2C family participated in ABA signal transduction (Park et al., 2009; He et al., 2019). Furthermore, as another important hormone, MeJA participates in stomatal closure and induces the expression of SnRK2.6 in *A. thaliana* (Mustilli et al., 2002). In the study, most BsPYLs, BsPP2Cs, and BsSnRK2s contained MeJA response elements, which indicated that they could participate in MeJA signal transduction to perform functions. The existence of various *cis*-acting elements associated with the responses of abiotic stresses and environment adaptation also revealed the importance of BsPYL-PP2C-SnRK2s in multiple biological processes and defense responses, which was consistent with previous studies (Ma et al., 2009; Park et al., 2009; Kulik et al., 2011; Hsu et al., 2021). The expression patterns of two randomly selected BsPYLs, four BsSnRK2s, and ten BsPP2Cs under various treatments, showed significant differences in expression, which suggested that BsPYL-PP2C-SnRK2s participated in multiple different signal transduction pathways in response to various stresses (Figure 5). In view of the important relationship between the PYL-PP2C-SnRK2s family and ABA signaling pathway, we focused on the analysis of gene expressions under ABA treatment. All 16 genes demonstrated varying degrees of responses to ABA signaling. For example, BsPYL6, BsPP2C22, and BsSnRK2.1 showed similar expression profiles under the ABA treatments. Their expression levels gradually increased with the extension of treatment times, which indicated that they likely interacted to participate in various biological processes related to ABA signal transduction. Interestingly, as a pair of paralogous genes, *BsPP2C6* and *BsPP2C22* had the opposite expression profiles under multiple treatments and stresses, while they showed similar expression patterns in different tissues and different germination stages. We speculated that they may be functionally complementary in *B. striata*. As another important member of group A, BsPP2C38 also responded to multiple treatments and stresses, and showed significantly high expression in BS3, which an important stage in seed germination of *B. striata*. These were consistent with previous studies that group A of PP2Cs was the most strongly related to ABA signal transduction for the regulation of multiple biological processes in *A. thaliana* (Allen et al., 1999; Saez et al., 2004; Umezawa et al., 2009; Sun et al., 2011).

To understand the interactions between BsPYLs and BsPP2Cs, as well as BsPP2Cs and BsSnRK2s, group A of BsPP2Cs was selected to conduct the Y2H and BiFC assays (Figures 8, 9). As a pair of paralogous genes, the BsPP2C20 and BsPP2C64 showed robust transactivation activity, which could not be effectively restrained through the addition of 3AT. According to the results, BsPP2C22 and BsPP2C38 interacted with multiple BsPYLs and BsSnRK2s proteins. This was consistent with earlier research, where AHG1 interacted with and was regulated by PYL9, PYL7, and PYL8, while all of the PYLs regulated HAI1, HAI2, and HAI3 in *A. thaliana* (Tischer et al., 2017). It was also suggested that ABI1 and ABI2 interacted with multiple PYLs in *A. thaliana* (Ma et al., 2009); ZmPP2C1/2/6/7/11/12/14/16 interacted with multiple ZmPYLs, while other ZmPP2Cs engaged in highly selective interactions with ZmPYLs. ZmPP2C1/6/7/14 revealed strong interactions with multiple ZmSnRK2s, and ZmPP2C2/11/12/16 showed highly selective interactions with ZmSnRK2s, while ZmPP2C3/4/8/9/15 exhibited weak interactions with ZmSnRK2s (Wang et al., 2018). This suggested that clade A of BsPP2Cs was essential for ABA signal transduction in *B. striata*, where BsPP2C22 and BsPP2C38 had highly redundant and robust interactions with BsPYLs and BsSnRK2s to respond to various biological processes and stresses.

## Conclusion

For this study, a total of nine BsPYLs, 66 BsPP2Cs, and seven BsSnRK2s genes were identified and characterized using *B. striata* genome databases. Bioinformatics analyses were initially completed, encompassing gene structures, protein physicochemical properties, conserved domains, *cis*-acting elements, and motifs. Phylogenetic analysis revealed that the BsPYL, BsPP2C, and BsSnRK2 families were classified into three, ten, and three subgroups, respectively. A total of 73 genes were localized on 16 pseudochromosomes, and 29 pairs of paralogous genes were identified via phylogenetic trees and intraspecific collinearity analyses. The expression profiles in different tissues and germination stages were different, as well as two BsPYLs, 10 BsPP2Cs, and four BsSnRK2s genes exhibited difference in response to multiple abiotic stresses. Additionally, subcellular localization analysis revealed that BsPP2C22, BsPP2C38, BsPP2C64, BsPYL2, BsPYL8, and BsSnRK2.4 were localized in the nucleus and membrane, while BsSnRK2.2 was localized in the nucleus. The results of Y2H and BiFC assays suggested that two important members of clade A (BsPP2C22 and BsPP2C38) could interact with BsPYLs and BsSnRK2s. To the best of our knowledge, this study comprises the first report on the identification and characterization of the PYL-PP2C-SnRK2s family in Orchidaceae. This work assists with further elucidating the core ABA regulation network in *B. striata*, while providing a conceptual basis for the intensive study of the regulation of seed germination and stress responses for *B. striata*.

## Data availability statement

The raw data supporting the conclusions of this article will be made available by the authors, without undue reservation.

## Author contributions

SL, DW, JN, and SW: conceptualization. SL, RZ, and CL: methodology. SL: writing – original draft. SL, RZ, and GJ: formal analysis and data curation. SL, YC, and CL: resources. GJ, RZ, and YC: investigation. JN, SW, DW, and ZW: funding acquisition. JN and ZW: writing – reviewing and editing. All authors contributed to the study conception and design and read and approved the final manuscript.

## Footnotes

1
https://www.arabidopsis.org/

2
https://phytozome.jgi.doe.gov/pz/portal.html

3
http://pfam.janelia.org

4
http://www.ebi.ac.uk/interpro/search/sequence-search

5
http://meme-suite.org/tools/meme

6
https://www.ncbi.nlm.nih.gov/Structure/bwrpsb/bwrpsb.cgi

7
http://smart.embl.de/

8
http://weblogo.berkeley.edu/logo.cgi

9
http://bioinformatics.psb.ugent.be/webtools/plantcare/html/

10
http://web.expasy.org/compute/pi/

11
https://services.healthtech.dtu.dk/service.php?SignalP-5.0

12
https://services.healthtech.dtu.dk/service.php?TMHMM-2.0

13
https://web.expasy.org/cgi-bin/protscale/protscale.pl

14
https://npsa-prabi.ibcp.fr/cgi-bin/npsa/automat.pl?page=npsa/sopma.html

15
https://swissmodel.expasy.org/interactive

16
https://www.ebi.ac.uk/Tools/msa/clustalo/

17
http://www.bork.embl.de/pal2nal/

18
https://www.genscript.com/tools/real-time-pcr-taqman-primer-design-tool

## Supplementary material

Supplementary Figure 1 The sequence logo of conserved motifs and the multiple sequence alignments of the BsPP2C **(A)**, BsPYL **(B)**, and BsSnRK2 **(C)** proteins conserved domain.

Supplementary Figure 2 Predicted signal peptide of the BsPP2C **(A)**, BsPYL **(B)**, and BsSnRK2 **(C)** proteins in *B. striata*.

Supplementary Figure 3 Predicted transmembrane segments of the BsPYL-PP2C-SnRK2s proteins in *B. striata*.

Supplementary Figure 4 Predicted hydrophobicity/hydrophilicity of the BsPYL-PP2C-SnRK2s proteins in *B. striata*. Negative values and positive values stand separately for hydrophilic and hydrophobic amino acid.

Supplementary Figure 5 Predicted tertiary structure of BsPP2Cs **(A)**, BsPYLs **(B)**, and BsSnRK2s **(C)** proteins in *B. striata*.

Supplementary Figure 6 *Cis*-acting elements of BsPP2Cs **(A)**, BsPYLs **(B)**, and BsSnRK2s **(C)** genes. The different colored boxes represent various response elements.

Supplementary Figure 7 Expression profiles of selected two BsPYLs, ten BsPP2Cs, and four BsSnRKs genes in response to different stress treatments for 0, 1, 3, 6, and 12 h, including ABA, SA, low temperature, wounding, CuSO_4_, and AgNO_3_. Data are means ± standard deviation. Asterisks (**p* ≤ 0.05; ***p* ≤ 0.01) indicate significant differences compared with different treatment times.
