# Supplementary material for: Comprehensive functional analysis of the PYL-PP2C-SnRK2s family in Bletilla striata reveals that BsPP2C22 and BsPP2C38 interact with BsPYLs and BsSnRK2s in response to multiple abiotic stresses
Source: Front Plant Sci. 2022 Aug 11;13:963069. doi: 10.3389/fpls.2022.963069 (PMC9404246; doi:10.3389/fpls.2022.963069)
Supplement: Supplementary file 1 [file Data_Sheet_1.docx]

Table S1 Primers for qRT-PCR of BsPYL-PP2C-SnRK2s gene family in *B. striata*

| **Gene** | Primers | Sequences（5’-3’） |
| --- | --- | --- |
| *BsPYL1* | *BsPYL1* F | TCGACGATGACCGCCATATT |
|  | *BsPYL1* R | ATTCCCTTCAGGCACATCCA |
| *BsPYL2* | *BsPYL2* F | AAGGTCACCGGATTCACCAT |
|  | *BsPYL2* R | CATCTTCCGTGTTCCCTTCG |
| *BsPYL3* | *BsPYL3* F | CCCATTCACCTCGTTTGGTC |
|  | *BsPYL3* R | CTCCAGCCTCTCTGTACTGG |
| *BsPYL4* | *BsPYL4* F | GGAGAGAATGCGGATCATGC |
|  | *BsPYL4* R | TTCGTATTCCTCCGGCGTTA |
| *BsPYL5* | *BsPYL5* F | GATAAACCGCAGGCCTACAA |
|  | *BsPYL5* R | CGGTAAGCCGGAGATTATGC |
| *BsPYL6* | *BsPYL6* F | CGTGCCTGAAGGAAACACAA |
|  | *BsPYL6* R | GGCACTGTGTGATCTTCCAC |
| *BsPYL7* | *BsPYL7* F | TCCACCCTCATCAAGCACAT |
|  | *BsPYL7* R | TTCCCTAACGCTTCCAACCT |
| *BsPYL8* | *BsPYL8* F | GCTCAGCTTTCGAGTCATCG |
|  | *BsPYL8* R | CGGCACATCCACCACATAAG |
| *BsPYL9* | *BsPYL9* F | GGAAACAGGCAGTTGGAGTC |
|  | *BsPYL9* R | GCGGCTGATCAAATCTCCTC |
| *BsPP2C1* | *BsPP2C1* F | TGGAGGACGAGCCGAGCATA |
|  | BsPP2C1 R | AGTTGGAAGTCGCCGAAGGC |
| *BsPP2C2* | BsPP2C2 F | CCTCGGCACCTTTCGGTCAT |
|  | BsPP2C2 R | GCTCGTCCGATGAAGAACGC |
| *BsPP2C3* | BsPP2C3 F | GGAGAAGACGATGGTGCGAT |
|  | BsPP2C3 R | CAAGCCCTCGAGGTATAGCG |
| *BsPP2C4* | BsPP2C4 F | GCCGGCGTCGGTAGAAGAG |
|  | BsPP2C4 R | CTAAGGCCTCCACCGCTGAC |
| *BsPP2C5* | BsPP2C5 F | CAAGAGCATTCGGGGATCGT |
|  | BsPP2C5 R | CCTTTGACGGCATCAACGA |
| *BsPP2C6* | BsPP2C6 F | AACGGGCGGACTTTTAGGAG |
|  | BsPP2C6 R | AGAGCTGTCGTCGTGTTGAG |
| *BsPP2C7* | BsPP2C7 F | CCGTCGGCTCTCTCGATTAC |
|  | BsPP2C7 R | CGAAAAGCAGTCCGTTGAGC |
| *BsPP2C8* | BsPP2C8 F | TGAACCGTATGCAGTGGAGG |
|  | BsPP2C8 R | GTACCGAAATGCCCTCACCA |
| *BsPP2C9* | BsPP2C9 F | CGGGAGCTTACGAGCGAACA |
|  | BsPP2C9 R | GCGCTGGCACTCTGTCTTGA |
| *BsPP2C10* | BsPP2C10 F | CAAATCCGGAAGGCGATCCT |
|  | BsPP2C10 R | TCCCTCGAAGGAGATGACGA |
| *BsPP2C11* | BsPP2C11 F | AAGATCGTCAGCCCCTGTTG |
|  | BsPP2C11 R | AAGCGGACCGGACTCTATCT |
| *BsPP2C12* | BsPP2C12 F | GGCTTGCTGCTCTCAGTTTG |
|  | BsPP2C12 R | CAAGCTCATGCCCCTTGACT |
| *BsPP2C13* | BsPP2C13 F | GGAATTGCGTCAGCACCTTC |
|  | BsPP2C13 R | AAGGCTCTCGGAAGAACTGC |
| *BsPP2C14* | BsPP2C14 F | ATGGCCAGCATGCTTATGGT |
|  | BsPP2C14 R | CAGACGTATCGAGCCACCTC |
| *BsPP2C15* | BsPP2C15 F | GCTGTTCCCCAATCTCCACA |
|  | BsPP2C15 R | TATCACCCCAACGAGGCAAC |
| *BsPP2C16* | BsPP2C16 F | ACAAAGCCCTGGCGTGGAAA |
|  | BsPP2C16 R | CCCACCCTCAGCACAGTTCC |
| *BsPP2C17* | BsPP2C17 F | TCGCCATCTTTGTCGTGGAA |
|  | BsPP2C17 R | TCTTCACACCCAACGCTACC |
| *BsPP2C18* | BsPP2C18 F | TCGCCGTTGTTCAGGCTAAT |
|  | BsPP2C18 R | GCTCCGAAGCAAACTTGTGG |
| *BsPP2C19* | BsPP2C19 F | AGCCACGGATGGTGTTTGGG |
|  | BsPP2C19 R | TGCGATGCCTCGCCTCTTAC |
| *BsPP2C20* | BsPP2C20 F | CCCTCTCCGACGATCACAAG |
|  | BsPP2C20 R | TTGTCACCACGTCCCATAGC |
| *BsPP2C21* | BsPP2C21 F | TCGCATCCTAGGGGTGCTAT |
|  | BsPP2C21 R | ATCTTTGCGGCTTGTCCTGA |
| *BsPP2C22* | BsPP2C22 F | TGGGTCCCGTGTATTTGGTG |
|  | BsPP2C22 R | TCGAGCAACATCACATGCCT |
| *BsPP2C23* | BsPP2C23 F | ATTTCGGAGCGGGCTCGTTT |
|  | BsPP2C23 R | CGGCTCTGCACTGAAGCGTA |
| *BsPP2C24* | BsPP2C24 F | GCTGGAACCGATGGGCTGTT |
|  | BsPP2C24 R | AGTCACCTGAGGGCCCAGTC |
| *BsPP2C25* | BsPP2C25 F | ACATGGCGTGTTGGTGGTGT |
|  | BsPP2C25 R | TCCCACAGTCCATCACTTGCG |
| *BsPP2C26* | BsPP2C26 F | TGTTGCAGCAGAGACGATG |
|  | BsPP2C26 R | GCGTCCTGATTAACCCCCTT |
| *BsPP2C27* | BsPP2C27 F | ATGGAGGTTGAGGGCAATGG |
|  | BsPP2C27 R | GAGACAAATTCCACCGCTGC |
| *BsPP2C28* | BsPP2C28 F | TAGGTCCTGGGGGTTCAACT |
|  | BsPP2C28 R | TGTCACAAAACCACCTCGCT |
| *BsPP2C29* | BsPP2C29 F | TTTGATGGTCATGGAGGCCC |
|  | BsPP2C29 R | CCAAAGATCAATGCGGCGAG |
| *BsPP2C30* | BsPP2C30 F | GGCACTCCTTCTGCTACGTT |
|  | BsPP2C30 R | AGCTTGAGGCGGAGTTATCG |
| *BsPP2C31* | BsPP2C31 F | GTTCCGGGACATTGGGCGAT |
|  | BsPP2C31 R | CCCGGACTCGATCTGGCTCT |
| *BsPP2C32* | BsPP2C32 F | GGGGAGGAACTTGCTGTGTT |
|  | BsPP2C32 R | TCCAAACACCTCTGCGACAA |
| *BsPP2C33* | BsPP2C33 F | GCTGGCGGCCATCTTCTGAT |
|  | BsPP2C33 R | TCACATGCTGCCCGAAGTCC |
| *BsPP2C34* | BsPP2C34 F | CCCACAAGAACGTGCCCGAA |
|  | BsPP2C34 R | AGTGCCGATCACCAAAGGCT |
| *BsPP2C35* | BsPP2C35 F | TTACGGCGACGAGCTTTTCT |
|  | BsPP2C35 R | TGATGAATCGAGAGGCGTCG |
| *BsPP2C36* | BsPP2C36 F | CTGGTTACCGCATCTGATGGA |
|  | BsPP2C36 R | GCTGCCAAATTGTCCATGC |
| *BsPP2C37* | BsPP2C37 F | GCCGCCGAAACCGAAACTTG |
|  | BsPP2C37 R | GAAGGCCTGCCGGGAGAATC |
| *BsPP2C38* | BsPP2C38 F | CGGCGAAGACGAATCAGAGA |
|  | BsPP2C38 R | TTCAACACCCGTAACCTCCG |
| *BsPP2C39* | BsPP2C39 F | CGACAGCATGCATTGTCGCT |
|  | BsPP2C39 R | ACTGGAGAGCGGAGGATGGT |
| *BsPP2C40* | BsPP2C40 F | GGAAGGAAGCCTGCATCAGT |
|  | BsPP2C40 R | AGTTCCCAATACCGCCCTTG |
| *BsPP2C41* | BsPP2C41 F | CTGCGGCCGAAGCTTAGATA |
|  | BsPP2C41 R | CCATGGCCGTCATATACCCC |
| *BsPP2C42* | BsPP2C42 F | CTCTGCTGATCCCGCCAAGG |
|  | BsPP2C42 R | GCGATTGCCGAGCAACGAAG |
| *BsPP2C43* | BsPP2C43 F | CGATGGGTTGTGTGCAGGGA |
|  | BsPP2C43 R | GACCTCGACGACAGCAGAGC |
| *BsPP2C44* | BsPP2C44 F | AGGATCGAAAGCCTTGGTGG |
|  | BsPP2C44 R | CAATGGCCTCCTGGTTGCTA |
| *BsPP2C45* | BsPP2C45 F | CATGGGCTGCTGCTCTCAGT |
|  | BsPP2C45 R | CCATCGGATGATTGGCCTTCCC |
| *BsPP2C46* | BsPP2C46 F | GGGATGCAGGACCGAGAAGC |
|  | BsPP2C46 R | CGATGGCAGTCGGGCTTGAT |
| *BsPP2C47* | BsPP2C47 F | GCGGAGCGAGAAGGATGAAT |
|  | BsPP2C47 R | CTTCCTCGAGCAATGGCAAG |
| *BsPP2C48* | BsPP2C48 F | TGAGGATGCTGGAGGAGTGGT |
|  | BsPP2C48 R | GCAGCCGATTACCAAACGCA |
| *BsPP2C49* | BsPP2C49 F | AATGCGAGGCTGCGAGTGAA |
|  | BsPP2C49 R | TGGCGCTCGAATCTGCCATC |
| *BsPP2C50* | BsPP2C50 F | GCTGGCGGCCGAATGGAAAT |
|  | BsPP2C50 R | GGCTCTGGTCCTCGAGCAAG |
| *BsPP2C51* | BsPP2C51 F | TCTGATGGACTCTGGGAGCA |
|  | BsPP2C51 R | GTGAAAGTGTCGACGGACCT |
| *BsPP2C52* | BsPP2C52 F | TGTCAGGCAAGCGAATTAGCCA |
|  | BsPP2C52 R | TGGCCCAAGTGGCCATCAAA |
| *BsPP2C53* | BsPP2C53 F | GCGGTTCGAGCATGGAGGTT |
|  | BsPP2C53 R | TGGCTCCGGCTTATCTCCGT |
| *BsPP2C54* | BsPP2C54 F | GCTTTGGAGCTCACACACAA |
|  | BsPP2C54 R | CGGTGCGTGTTTCATACTGT |
| *BsPP2C55* | BsPP2C55 F | GACTCGAGGGCTGTGCTTGG |
|  | BsPP2C55 R | CTCACCTCCTCCACCGCAAC |
| *BsPP2C56* | BsPP2C56 F | AGCTGTCATTTGTCGGGGAG |
|  | BsPP2C56 R | GCTTCAAGAGACGGTCACCA |
| *BsPP2C57* | BsPP2C57 F | ATTGCCAATCCATGCGAAGC |
|  | BsPP2C57 R | ACTTTCTTCGACCGCTGGTT |
| *BsPP2C58* | BsPP2C58 F | TGAAGGATGCTGATGGCCTG |
|  | BsPP2C58 R | CCTCCTTTTGAGTGCCTCGT |
| *BsPP2C59* | BsPP2C59 F | TCTTACCGCAGAGCCGGAGT |
|  | BsPP2C59 R | TCGGAGTCCTCTTCGCACCA |
| *BsPP2C60* | BsPP2C60 F | GGGTTTGAGGCGCTGGTCAT |
|  | BsPP2C60 R | CGACTCGGCCATCAACGTCA |
| *BsPP2C61* | BsPP2C61 F | AAATGGCGAGATCGGAAGGG |
|  | BsPP2C61 R | CGCATTTGCCACCATAGAGC |
| *BsPP2C62* | BsPP2C62 F | TGCACAGCGTGTGTGGCTAT |
|  | BsPP2C62 R | ACCTCGAGCTCTGGCTTGTG |
| *BsPP2C63* | BsPP2C63 F | GTAACTGGCGTTTTTGGCGT |
|  | BsPP2C63 R | CGGCCGGTGATCAGAAGTAA |
| *BsPP2C64* | BsPP2C64 F | ATGATATGAAGCCGGAGGCG |
|  | BsPP2C64 R | GCATCCGATTCTTGCACCAC |
| *BsPP2C65* | BsPP2C65 F | GAACTGCATCGGCGTGCTTG |
|  | BsPP2C65 R | GCTGAATGCCGCCCAATGTC |
| *BsPP2C66* | BsPP2C66 F | GGACCTTTGCAAGGACGACGA |
|  | BsPP2C66 R | GCACACGGCAGAGAGGCTAC |
| *BsSnRK2.1* | *BsSnRK2.1* F | TGCCAAGAGAGCTGACAGAA |
|  | *BsSnRK2.1* R | TAGCCCAGCATGGACTTTGA |
| *BsSnRK2.2* | *BsSnRK2.2* F | GAGGCTTATGCGGAACAAGG |
|  | *BsSnRK2.2* R | ATCGCAAGATCAGTCGGAGT |
| *BsSnRK2.3* | *BsSnRK2.3* F | CGATCAGCCAATGCAGAACA |
|  | *BsSnRK2.3* R | TTCTCCGCTGCTATCGACAT |
| *BsSnRK2.4* | *BsSnRK2.4* F | GATGGCAAGATTGCGGATGT |
|  | *BsSnRK2.4* R | TCGACCGATTGTCTTTCGGA |
| *BsSnRK2.5* | *BsSnRK2.5* F | TGTATCTCCCGAGTGCAGAC |
|  | *BsSnRK2.5* R | ATCGATGCTTTGCATTGGCT |
| *BsSnRK2.6* | *BsSnRK2.6* F | GGAGGTCTTCTTGACGCCTA |
|  | *BsSnRK2.6* R | CATCTTCACCGAACCTCCCT |
| *BsSnRK2.7* | *BsSnRK2.7* F | TTGCAGATGTCTGGTCCTGT |
|  | *BsSnRK2.7* R | CAACAAAGATGCGGGAGAGG |

Table S2 Primers for cloning BsPYLs, BsPP2Cs and BsSnRK2s in *B. striata*

| **Primers** | **Sequences（5’-3’）** |
| --- | --- |
| *BsPYL2* F | ATGGACCGCAGCGGAGCACA |
| *BsPYL2* R | TCATTTCCCCATCTCCAGAATCCCT |
| *BsPYL3* F | ATGTTAGGGGAGAGCAGAGCAAGG |
| *BsPYL3* R | TCAGGCTTGATATCCCTGGATGG |
| *BsPYL6* F | ATGGTGGGAGAGAGCAGAGCAAGA |
| *BsPYL6* R | TCACAGATCAATGGGCACTGTGTG |
| *BsPYL8* F | ATGCCTTACACAACTCCCCCAAAATCCTC |
| *BsPYL8* R | TCACGCCGCCGGCCGACCACT |
| *BsPP2C22* F | ATGGAGGAGAGTTCTCTTGTGC |
| *BsPP2C22* R | TTATGTTTTGCTCTTGAACTTCCTG |
| *BsPP2C20* F | ATGGCGGAGATATGCTGCGGAGT |
| *BsPP2C20* R | TCACAATTTCCTCCTAAGATCGACCAC |
| *BsPP2C64* F | ATGGCCGAGATATGCTTGGAAATCG |
| *BsPP2C64* R | CTATAATTTCCTCCTTAGATCCACAACAAC |
| *BsPP2C38* F | ATGGTTGATGTGTACGTAGATATGTTCGG |
| *BsPP2C38* R | TTATCGGTGCTTCTCCTTCCCAGC |
| *BsSnRK2.1* F | ATGGAGAAATACGAGATGGTGC |
| *BsSnRK2.1* R | TCATTGGAGATCAAAGGTATAACTC |
| *BsSnRK2.2* F | ATGGAGAAGTATGAGATGGTGAA |
| *BsSnRK2.2* R | TCAACTTATTTGGTAGTCACCACT |
| *BsSnRK2.4* F | ATGGAGGCGAGATATGAGCCTT |
| *BsSnRK2.4* R | TCAAACGAGTGCCAAGAAATCA |
| *BsSnRK2.5* F | ATGGATCGTGCGGCAATGAC |
| *BsSnRK2.5* R | TCACATGGCATAAACTATCTCCCC |
| *BsSnRK2.7* F | ATGGACAAGTACGAGGTAGTGAAG |
| *BsSnRK2.7* R | TTATATCTCCGAGTCCTTTCGATCAAC |

Table S3 Primers without termination codon for subcellular localization vectors of *BsPYLs*, *BsPP2Cs* and *BsSnRK2s* in *B. striata*

| **Primers** | **Sequences（5’-3’）** |
| --- | --- |
| attB-*BsPYL2* F | GGGGACAAGTTTGTACAAAAAAGCAGGCTTC  ATGGACCGCAGCGGAGCACA |
| attB-*BsPYL2* R | GGGGACCACTTTGTACAAGAAAGCTGGGTC  TTTCCCCATCTCCAGAATCCCT |
| attB-*BsPYL8* F | GGGGACAAGTTTGTACAAAAAAGCAGGCTTC  ATGCCTTACACAACTCCCCCAAAATCCTC |
| attB-*BsPYL8* R | GGGGACCACTTTGTACAAGAAAGCTGGGTC  CGCCGCCGGCCGACCACT |
| attB-*BsPP2C22* F | GGGGACAAGTTTGTACAAAAAAGCAGGCTTC  ATGGAGGAGAGTTCTCTTGTGC |
| attB-*BsPP2C22* R | GGGGACCACTTTGTACAAGAAAGCTGGGTC  TGTTTTGCTCTTGAACTTCCTG |
| attB-*BsPP2C38* F | GGGGACAAGTTTGTACAAAAAAGCAGGCTTC  ATGGTTGATGTGTACGTAGATATGTTCGG |
| attB-*BsPP2C38* R | GGGGACCACTTTGTACAAGAAAGCTGGGTC  TCGGTGCTTCTCCTTCCCAGC |
| attB-*BsPP2C64* F | GGGGACAAGTTTGTACAAAAAAGCAGGCTTC  ATGGCCGAGATATGCTTGGAAATCG |
| attB-*BsPP2C64* R | GGGGACCACTTTGTACAAGAAAGCTGGGTC  TAATTTCCTCCTTAGATCCACAACAAC |
| attB-*BsSnRK2.2* F | GGGGACAAGTTTGTACAAAAAAGCAGGCTTC  ATGGAGAAGTATGAGATGGTGAA |
| attB-*BsSnRK2.2* R | GGGGACCACTTTGTACAAGAAAGCTGGGTC  ACTTATTTGGTAGTCACCACT |
| attB-*BsSnRK2.4* F | GGGGACAAGTTTGTACAAAAAAGCAGGCTTC  ATGGAGGCGAGATATGAGCCTT |
| attB-*BsSnRK*2.4 R | GGGGACCACTTTGTACAAGAAAGCTGGGTC  AACGAGTGCCAAGAAATCA |

Table S4 Primers with termination codon for yeast two hybrid vectors of BsPYLs, BsPP2Cs and BsSnRK2s in *B. striata*

| **Primers** | **Sequences（5’-3’）** |
| --- | --- |
| attB-*BsPYL2* F | GGGGACAAGTTTGTACAAAAAAGCAGGCTTC  ATGGACCGCAGCGGAGCACA |
| attB-*BsPYL2* R | GGGGACCACTTTGTACAAGAAAGCTGGGTC  TCATTTCCCCATCTCCAGAATCCCT |
| attB-*BsPYL3* F | GGGGACAAGTTTGTACAAAAAAGCAGGCTTC  ATGTTAGGGGAGAGCAGAGCAAGG |
| attB-*BsPYL3* R | GGGGACCACTTTGTACAAGAAAGCTGGGTC  TCAGGCTTGATATCCCTGGATGG |
| attB-*BsPYL6* F | GGGGACAAGTTTGTACAAAAAAGCAGGCTTC  ATGGTGGGAGAGAGCAGAGCAAGA |
| attB-*BsPYL6* R | GGGGACCACTTTGTACAAGAAAGCTGGGTC  TCACAGATCAATGGGCACTGTGTG |
| attB-*BsPYL8* F | GGGGACAAGTTTGTACAAAAAAGCAGGCTTC  ATGCCTTACACAACTCCCCCAAAATCCTC |
| attB-*BsPYL8* R | GGGGACCACTTTGTACAAGAAAGCTGGGTC  TCACGCCGCCGGCCGACCACT |
| attB-*BsPP2C20* F | GGGGACAAGTTTGTACAAAAAAGCAGGCTTC  ATGGCGGAGATATGCTGCGGAGT |
| attB-*BsPP2C20* R | GGGGACCACTTTGTACAAGAAAGCTGGGTC  TCACAATTTCCTCCTAAGATCGACCAC |
| attB-*BsPP2C22* F | GGGGACAAGTTTGTACAAAAAAGCAGGCTTC  ATGGAGGAGAGTTCTCTTGTGC |
| attB-*BsPP2C22* R | GGGGACCACTTTGTACAAGAAAGCTGGGTC  TTATGTTTTGCTCTTGAACTTCCTG |
| attB-*BsPP2C38* F | GGGGACAAGTTTGTACAAAAAAGCAGGCTTC  ATGGTTGATGTGTACGTAGATATGTTCGG |
| attB-*BsPP2C38* R | GGGGACCACTTTGTACAAGAAAGCTGGGTC  TTATCGGTGCTTCTCCTTCCCAGC |
| attB-*BsPP2C64* F | GGGGACAAGTTTGTACAAAAAAGCAGGCTTC  ATGGCCGAGATATGCTTGGAAATCG |
| attB-*BsPP2C64* R | GGGGACCACTTTGTACAAGAAAGCTGGGTC  CTATAATTTCCTCCTTAGATCCACAACAAC |
| attB-*BsSnRK2.1* F | GGGGACAAGTTTGTACAAAAAAGCAGGCTTC  ATGGAGAAATACGAGATGGTGC |
| attB-*BsSnRK2.1* R | GGGGACCACTTTGTACAAGAAAGCTGGGTC  TCATTGGAGATCAAAGGTATAACTC |
| attB-*BsSnRK2.4* F | GGGGACAAGTTTGTACAAAAAAGCAGGCTTC  ATGGAGGCGAGATATGAGCCTT |
| attB-*BsSnRK2.4* R | GGGGACCACTTTGTACAAGAAAGCTGGGTC  TCAAACGAGTGCCAAGAAATCA |
| attB-*BsSnRK2.5* F | GGGGACAAGTTTGTACAAAAAAGCAGGCTTC  ATGGATCGTGCGGCAATGAC |
| attB-*BsSnRK2.5* R | GGGGACCACTTTGTACAAGAAAGCTGGGTC  TCACATGGCATAAACTATCTCCCC |
| attB-*BsSnRK2.7* F | GGGGACAAGTTTGTACAAAAAAGCAGGCTTC  ATGGACAAGTACGAGGTAGTGAAG |
| attB-*BsSnRK2.7* R | GGGGACCACTTTGTACAAGAAAGCTGGGTC  TTATATCTCCGAGTCCTTTCGATCAAC |

Table S5 Primers without termination codon for Bimolecular Fluorescence Complementation (BiFC) Assay vectors of BsPYLs, BsPP2Cs and BsSnRK2s in *B. striata*

| **Primers** | **Sequences（5’-3’）** |
| --- | --- |
| attB-*BsPYL2* F | GGGGACAAGTTTGTACAAAAAAGCAGGCTTC  ATGGACCGCAGCGGAGCACA |
| attB-*BsPYL2* R | GGGGACCACTTTGTACAAGAAAGCTGGGTC  TTTCCCCATCTCCAGAATCCCT |
| attB-*BsPYL3* F | GGGGACAAGTTTGTACAAAAAAGCAGGCTTC  ATGTTAGGGGAGAGCAGAGCAAGG |
| attB-*BsPYL3* R | GGGGACCACTTTGTACAAGAAAGCTGGGTC  GGCTTGATATCCCTGGATGG |
| attB-*BsPYL6* F | GGGGACAAGTTTGTACAAAAAAGCAGGCTTC  ATGGTGGGAGAGAGCAGAGCAAGA |
| attB-*BsPYL6* R | GGGGACCACTTTGTACAAGAAAGCTGGGTC  CAGATCAATGGGCACTGTGTG |
| attB-*BsPYL8* F | GGGGACAAGTTTGTACAAAAAAGCAGGCTTC  ATGCCTTACACAACTCCCCCAAAATCCTC |
| attB-*BsPYL8* R | GGGGACCACTTTGTACAAGAAAGCTGGGTC  CGCCGCCGGCCGACCACT |
| attB-*BsPP2C22* F | GGGGACAAGTTTGTACAAAAAAGCAGGCTTC  ATGGAGGAGAGTTCTCTTGTGC |
| attB-*BsPP2C22* R | GGGGACCACTTTGTACAAGAAAGCTGGGTC  TGTTTTGCTCTTGAACTTCCTG |
| attB-*BsPP2C38* F | GGGGACAAGTTTGTACAAAAAAGCAGGCTTC  ATGGTTGATGTGTACGTAGATATGTTCGG |
| attB-*BsPP2C38* R | GGGGACCACTTTGTACAAGAAAGCTGGGTC  TCGGTGCTTCTCCTTCCCAGC |
| attB-*BsSnRK2.1* F | GGGGACAAGTTTGTACAAAAAAGCAGGCTTC  ATGGAGAAATACGAGATGGTGC |
| attB-*BsSnRK2.1* R | GGGGACCACTTTGTACAAGAAAGCTGGGTC  TTGGAGATCAAAGGTATAACTC |
| attB-*BsSnRK2.4* F | GGGGACAAGTTTGTACAAAAAAGCAGGCTTC  ATGGAGGCGAGATATGAGCCTT |
| attB-*BsSnRK2.4* R | GGGGACCACTTTGTACAAGAAAGCTGGGTC  AACGAGTGCCAAGAAATCA |
| attB-*BsSnRK2.5* F | GGGGACAAGTTTGTACAAAAAAGCAGGCTTC  ATGGATCGTGCGGCAATGAC |
| attB-*BsSnRK2.5* R | GGGGACCACTTTGTACAAGAAAGCTGGGTC  CATGGCATAAACTATCTCCCC |
| attB-*BsSnRK2.7* F | GGGGACAAGTTTGTACAAAAAAGCAGGCTTC  ATGGACAAGTACGAGGTAGTGAAG |
| attB-*BsSnRK2.7* R | GGGGACCACTTTGTACAAGAAAGCTGGGTC  TATCTCCGAGTCCTTTCGATCAAC |

Table S6 Gene features of BsPYL-PP2C-SnRK2s gene family in *B. striata*

| **Gene ID** | **Gene length(bp)** | **cDNA length(bp)** | **Protein(aa)** | **Mw(Da)** | **p*I*** |
| --- | --- | --- | --- | --- | --- |
| BsPYL1 | 708 | 708 | 235 | 25517.06 | 7.10 |
| BsPYL2 | 1134 | 642 | 213 | 23235.18 | 5.15 |
| BsPYL3 | 2448 | 660 | 219 | 24072.4 | 6.17 |
| BsPYL4 | 3122 | 855 | 284 | 30945.75 | 7.65 |
| BsPYL5 | 362 | 291 | 96 | 10119.58 | 9.24 |
| BsPYL6 | 3357 | 639 | 212 | 23671.73 | 579 |
| BsPYL7 | 4736 | 558 | 185 | 20931.84 | 6.06 |
| BsPYL8 | 970 | 645 | 214 | 22560.5 | 6.82 |
| BsPYL9 | 6196 | 765 | 254 | 28610.76 | 7.01 |
| BsPP2C1 | 6696 | 762 | 253 | 27699.17 | 5.67 |
| BsPP2C2 | 3019 | 1053 | 350 | 38452.59 | 5.50 |
| BsPP2C3 | 10584 | 1269 | 422 | 45617.48 | 7.88 |
| BsPP2C4 | 20841 | 1071 | 356 | 39088.89 | 4.86 |
| BsPP2C5 | 24191 | 858 | 285 | 31241.79 | 6.46 |
| BsPP2C6 | 31831 | 1833 | 610 | 66178.81 | 4.99 |
| BsPP2C7 | 3163 | 1587 | 528 | 57604.59 | 5.17 |
| BsPP2C8 | 15673 | 1311 | 436 | 47108.29 | 8.17 |
| BsPP2C9 | 57933 | 1311 | 436 | 47737.88 | 6.48 |
| BsPP2C10 | 3003 | 837 | 278 | 31719.89 | 4.72 |
| BsPP2C11 | 8798 | 1125 | 374 | 41304.39 | 9.12 |
| BsPP2C12 | 36271 | 855 | 284 | 31267.57 | 6.55 |
| BsPP2C13 | 12277 | 1323 | 440 | 47148.56 | 5.72 |
| BsPP2C14 | 6370 | 1182 | 393 | 43409.59 | 6.14 |
| BsPP2C15 | 9537 | 1257 | 418 | 46893.76 | 8.43 |
| BsPP2C16 | 29493 | 2781 | 926 | 102937.06 | 5.93 |
| BsPP2C17 | 2521 | 1203 | 400 | 44108.34 | 5.69 |
| BsPP2C18 | 8539 | 1122 | 373 | 42225.14 | 7.00 |
| BsPP2C19 | 6077 | 1098 | 365 | 40212.81 | 7.88 |
| BsPP2C20 | 1908 | 1260 | 419 | 45307.32 | 5.40 |
| BsPP2C21 | 5478 | 1059 | 352 | 38262.95 | 5.43 |
| BsPP2C22 | 22924 | 1611 | 536 | 57285.81 | 4.68 |
| BsPP2C23 | 23468 | 1935 | 644 | 70521.09 | 6.39 |
| BsPP2C24 | 30224 | 1329 | 442 | 47800.94 | 6.50 |
| BsPP2C25 | 1174 | 432 | 143 | 15807.65 | 448 |
| BsPP2C26 | 3481 | 1377 | 458 | 50429.55 | 5.74 |
| BsPP2C27 | 3761 | 1086 | 361 | 40365.17 | 6.19 |
| BsPP2C28 | 11874 | 2757 | 918 | 33613.75 | 6.72 |
| BsPP2C29 | 2880 | 1215 | 404 | 44781.88 | 4.94 |
| BsPP2C30 | 9849 | 1845 | 614 | 67501.14 | 5.74 |
| BsPP2C31 | 52530 | 1113 | 370 | 41069.63 | 6.87 |
| BsPP2C32 | 3060 | 1218 | 405 | 43928.33 | 5.91 |
| BsPP2C33 | 1955 | 717 | 238 | 26142.8 | 6.29 |
| BsPP2C34 | 30197 | 705 | 234 | 25915.81 | 9.58 |
| BsPP2C35 | 7004 | 1143 | 380 | 42635.61 | 6.15 |
| BsPP2C36 | 15509 | 702 | 233 | 25331.3 | 6.00 |
| BsPP2C37 | 23070 | 1179 | 392 | 42969.21 | 6.62 |
| BsPP2C38 | 25673 | 1260 | 419 | 45114.18 | 7.87 |
| BsPP2C39 | 6257 | 852 | 283 | 31478.78 | 6.13 |
| BsPP2C40 | 2561 | 1023 | 340 | 37878.87 | 6.20 |
| BsPP2C41 | 7838 | 1095 | 364 | 40917.07 | 8.08 |
| BsPP2C42 | 37638 | 1134 | 377 | 41309.14 | 5.57 |
| BsPP2C43 | 33020 | 1788 | 595 | 63938.37 | 5.02 |
| BsPP2C44 | 1051 | 753 | 250 | 27482.11 | 5.10 |
| BsPP2C45 | 30274 | 852 | 283 | 31478.78 | 6.13 |
| BsPP2C46 | 68944 | 1290 | 429 | 46568.32 | 7.57 |
| BsPP2C47 | 5890 | 498 | 165 | 17898.36 | 5.66 |
| BsPP2C48 | 60699 | 753 | 250 | 27726.06 | 4.87 |
| BsPP2C49 | 3174 | 375 | 124 | 13915.18 | 9.97 |
| BsPP2C50 | 16644 | 1200 | 399 | 44427.78 | 6.94 |
| BsPP2C51 | 7698 | 1194 | 397 | 44144.49 | 8.74 |
| BsPP2C52 | 9100 | 855 | 284 | 31252.75 | 6.83 |
| BsPP2C53 | 11246 | 1281 | 426 | 46914.90 | 5.08 |
| BsPP2C54 | 54974 | 2298 | 765 | 84809.18 | 5.11 |
| BsPP2C55 | 4567 | 1134 | 377 | 41432.55 | 8.47 |
| BsPP2C56 | 38070 | 885 | 294 | 31739.16 | 4.98 |
| BsPP2C57 | 44408 | 1323 | 440 | 48208.82 | 6.43 |
| BsPP2C58 | 28166 | 1134 | 377 | 41082.53 | 5.50 |
| BsPP2C59 | 3369 | 1185 | 394 | 43261.74 | 4.73 |
| BsPP2C60 | 25028 | 1092 | 363 | 40174.95 | 5.39 |
| BsPP2C61 | 24178 | 675 | 224 | 24106.03 | 5.72 |
| BsPP2C62 | 56421 | 1071 | 356 | 39246.96 | 5.08 |
| BsPP2C63 | 1760 | 954 | 317 | 34818 | 5.59 |
| BsPP2C64 | 2602 | 1158 | 385 | 42360.63 | 6.43 |
| BsPP2C65 | 8143 | 1164 | 387 | 43223.37 | 6.47 |
| BsPP2C66 | 6785 | 1068 | 355 | 39117.93 | 5.09 |
| BsSnRK2.1 | 17669 | 1167 | 388 | 44632.28 | 5.23 |
| BsSnRK2.2 | 9460 | 1089 | 362 | 41823.45 | 5.24 |
| BsSnRK2.3 | 58560 | 1137 | 378 | 42874.12 | 4.97 |
| BsSnRK2.4 | 19244 | 1008 | 335 | 37810.16 | 5.69 |
| BsSnRK2.5 | 14654 | 1092 | 363 | 41175.98 | 4.89 |
| BsSnRK2.6 | 8087 | 1023 | 340 | 38758.23 | 5.44 |
| BsSnRK2.7 | 10916 | 1107 | 368 | 42306.72 | 9.28 |

Table S7 Secondary structure elements of BsPYL-PP2C-SnRK2s protein in *B. striata*

| **Protein** | **Alpha helix** | **Extended strand** | **Beta turn** | **Random coil** |
| --- | --- | --- | --- | --- |
| BsPYL1 | 31.91% | 21.70% | 5.53% | 40.85% |
| BsPYL2 | 37.09% | 15.49% | 3.76% | 43.66% |
| BsPYL3 | 42.47% | 15.53% | 5.94% | 36.07% |
| BsPYL4 | 30.63% | 20.42% | 9.15% | 39.79% |
| BsPYL5 | 35.42% | 12.50% | 5.21% | 46.88% |
| BsPYL6 | 34.91% | 19.34% | 4.72% | 41.04% |
| BsPYL7 | 41.08% | 18.38% | 5.41% | 35.14% |
| BsPYL8 | 31.31% | 15.89% | 4.67% | 48.13% |
| BsPYL9 | 41.34% | 18.50% | 7.87% | 32.28% |
| BsPP2C1 | 34.39% | 25.30% | 9.09% | 31.23% |
| BsPP2C2 | 36.29% | 15.14% | 7.43% | 41.14% |
| BsPP2C3 | 30.81% | 18.72% | 5.45% | 45.02% |
| BsPP2C4 | 42.42% | 19.10% | 7.02% | 31.46% |
| BsPP2C5 | 36.84% | 21.40% | 8.77% | 32.98% |
| BsPP2C6 | 36.07% | 16.23% | 6.39% | 41.31% |
| BsPP2C7 | 35.61% | 15.15% | 4.92% | 44.32% |
| BsPP2C8 | 34.17% | 17.89% | 6.19% | 41.74% |
| BsPP2C9 | 31.88% | 17.66% | 7.11% | 43.35% |
| BsPP2C10 | 41.73% | 17.27% | 6.47% | 34.53% |
| BsPP2C11 | 33.16% | 18.98% | 9.36% | 38.50% |
| BsPP2C12 | 34.51% | 21.83% | 7.39% | 36.27% |
| BsPP2C13 | 36.14% | 17.05% | 4.77% | 42.05% |
| BsPP2C14 | 35.37% | 17.56% | 8.40% | 38.68% |
| BsPP2C15 | 39.71% | 18.18% | 6.46% | 35.65% |
| BsPP2C16 | 32.40% | 14.04% | 5.83% | 47.73% |
| BsPP2C17 | 39.50% | 18.25% | 8.00% | 34.25% |
| BsPP2C18 | 32.44% | 21.45% | 8.04% | 38.07% |
| BsPP2C19 | 37.26% | 21.10% | 5.48% | 36.16% |
| BsPP2C20 | 36.04% | 14.08% | 4.30% | 45.58% |
| BsPP2C21 | 42.90% | 16.48% | 5.11% | 35.51% |
| BsPP2C22 | 34.33% | 15.11% | 4.85% | 45.71% |
| BsPP2C23 | 20.34% | 19.57% | 5.75% | 54.35% |
| BsPP2C24 | 34.62% | 16.97% | 5.88% | 42.53% |
| BsPP2C25 | 37.06% | 20.28% | 11.89% | 30.77% |
| BsPP2C26 | 32.97% | 18.78% | 6.99% | 41.27% |
| BsPP2C27 | 41.00% | 16.90% | 8.86% | 33.24% |
| BsPP2C28 | 35.74% | 19.02% | 7.21% | 38.03% |
| BsPP2C29 | 41.83% | 11.39% | 6.93% | 39.85% |
| BsPP2C30 | 33.22% | 12.05% | 5.37% | 49.35% |
| BsPP2C31 | 37.30% | 18.65% | 7.03% | 37.03% |
| BsPP2C32 | 35.84% | 17.54% | 8.02% | 38.60% |
| BsPP2C33 | 39.92% | 19.75% | 6.72% | 33.61% |
| BsPP2C34 | 40.60% | 23.08% | 7.69% | 28.63% |
| BsPP2C35 | 29.47% | 20.00% | 6.32% | 44.21% |
| BsPP2C36 | 27.47% | 19.74% | 12.02% | 40.77% |
| BsPP2C37 | 33.42% | 21.17% | 5.10% | 40.31% |
| BsPP2C38 | 31.98% | 13.84% | 3.82% | 50.36% |
| BsPP2C39 | 23.25% | 15.43% | 5.56% | 55.76% |
| BsPP2C40 | 30.88% | 22.65% | 6.47% | 40.00% |
| BsPP2C41 | 41.83% | 11.39% | 6.93% | 39.85% |
| BsPP2C42 | 38.46% | 19.10% | 5.04% | 37.40% |
| BsPP2C43 | 27.23% | 22.02% | 6.39% | 44.37% |
| BsPP2C44 | 45.60% | 20.80% | 10.00% | 23.60% |
| BsPP2C45 | 38.16% | 21.55% | 6.36% | 33.92% |
| BsPP2C46 | 31.93% | 18.88% | 6.29% | 42.89% |
| BsPP2C47 | 40.53% | 22.11% | 8.68% | 28.68% |
| BsPP2C48 | 39.20% | 20.00% | 10.40% | 30.40% |
| BsPP2C49 | 32.26% | 20.16% | 4.03% | 43.55% |
| BsPP2C50 | 31.93% | 18.88% | 6.29% | 42.89% |
| BsPP2C51 | 36.02% | 15.87% | 6.05% | 42.07% |
| BsPP2C52 | 38.73% | 20.77% | 7.04% | 33.45% |
| BsPP2C53 | 27.46% | 18.31% | 5.63% | 48.59% |
| BsPP2C54 | 37.52% | 19.61% | 6.01% | 36.86% |
| BsPP2C55 | 32.89% | 20.16% | 7.16% | 39.79% |
| BsPP2C56 | 36.73% | 21.09% | 7.48% | 34.69% |
| BsPP2C57 | 30.45% | 20.00% | 5.45% | 44.09% |
| BsPP2C58 | 41.11% | 10.08% | 5.57% | 43.24% |
| BsPP2C59 | 41.62% | 13.96% | 8.88% | 35.53% |
| BsPP2C60 | 35.54% | 21.49% | 7.99% | 34.99% |
| BsPP2C61 | 41.96% | 16.52% | 6.70% | 34.82% |
| BsPP2C62 | 37.08% | 16.85% | 5.34% | 40.73% |
| BsPP2C63 | 36.91% | 17.35% | 6.62% | 39.12% |
| BsPP2C64 | 37.40% | 17.14% | 7.27% | 38.18% |
| BsPP2C65 | 37.73% | 18.35% | 5.94% | 37.98% |
| BsPP2C66 | 41.50% | 14.97% | 6.80% | 36.73% |
| BsSnRK2.1 | 39.43% | 15.21% | 4.90% | 40.46% |
| BsSnRK2.2 | 42.54% | 14.64% | 4.70% | 38.12% |
| BsSnRK2.3 | 36.51% | 17.20% | 6.35% | 39.95% |
| BsSnRK2.4 | 35.52% | 15.22% | 3.28% | 45.97% |
| BsSnRK2.5 | 35.26% | 17.08% | 4.68% | 42.98% |
| BsSnRK2.6 | 32.94% | 17.06% | 6.47% | 43.53% |
| BsSnRK2.7 | 36.41% | 15.49% | 5.43% | 42.66% |

Table S8 Ka/Ks analysis of BsPYL-PP2C-SnRK2s paralogous in *B. striata*

| Gene pair | S | N | Ks | Ka | Ka/Ks |
| --- | --- | --- | --- | --- | --- |
| BsPYL2/4 | 148.2 | 490.8 | 1.0139 | 0.217 | 0.2141 |
| BsPYL5/8 | 41.5 | 246.5 | 9.1664 | 0.1316 | 0.0144 |
| BsPYL6/7 | 139 | 413 | 0.8542 | 0.0706 | 0.0826 |
| BsPP2C21/47 | 124.9 | 337.1 | 2.089 | 0.3736 | 0.1788 |
| BsPP2C20/64 | 283.3 | 853.7 | 3.134 | 0.291 | 0.0929 |
| BsPP2C61/38 | 169 | 503 | 43.186 | 2.8599 | 0.0662 |
| BsPP2C6/22 | 416.7 | 1185.3 | 3.6986 | 0.4618 | 0.1249 |
| BsPP2C27/17 | 257.3 | 807.7 | 0.6376 | 0.1886 | 0.2957 |
| BsPP2C63/32 | 226.5 | 715.5 | 1.2745 | 0.1563 | 0.1226 |
| BsPP2C56/25 | 116.1 | 312.9 | 0.7176 | 0.059 | 0.0822 |
| BsPP2C48/60 | 87 | 285 | 1.3907 | 0.5473 | 0.3936 |
| BsPP2C52/5 | 209.3 | 642.7 | 0.8714 | 0.1007 | 0.1156 |
| BsPP2C45/12 | 204.3 | 644.7 | 0.5416 | 0.1402 | 0.2589 |
| BsPP2C62/66 | 210.7 | 749.3 | 0.7044 | 0.1159 | 0.1646 |
| BsPP2C29/59 | 331.1 | 832.9 | 1.2128 | 0.1366 | 0.1126 |
| BsPP2C3/46 | 332.2 | 924.8 | 48.5889 | 0.2161 | 0.0044 |
| BsPP2C8/9 | 334.4 | 958.6 | 0.5117 | 0.1011 | 0.1975 |
| BsPP2C43/54 | 419.4 | 1248.6 | 49.8501 | 0.6209 | 0.0125 |
| BsPP2C10/7 | 194.8 | 588.2 | 2.6363 | 0.1704 | 0.0646 |
| BsPP2C18/55 | 288.1 | 806.9 | 1.1313 | 0.1626 | 0.1437 |
| BsPP2C41/11 | 274.3 | 802.7 | 2.3304 | 0.2479 | 0.1064 |
| BsPP2C51/14 | 275.4 | 894.6 | 0.5587 | 0.1632 | 0.2921 |
| BsPP2C50/33 | 180.9 | 524.1 | 1.4482 | 0.1675 | 0.1157 |
| BsPP2C36/37 | 137.6 | 489.4 | 0.3426 | 0.2128 | 0.6211 |
| BsPP2C40/1 | 195.4 | 554.6 | 2.9386 | 0.2507 | 0.0853 |
| BsPP2C39/24 | 281.6 | 1041.4 | 4.5982 | 0.4401 | 0.0957 |
| BsSnRK2.2/2.7 | 269.9 | 816.1 | 0.9501 | 0.1278 | 0.1346 |
| BsSnRK2.3/2.5 | 294.8 | 788.2 | 0.7476 | 0.0526 | 0.0703 |
