# Supplementary figures and images for: Comprehensive functional analysis of the PYL-PP2C-SnRK2s family in Bletilla striata reveals that BsPP2C22 and BsPP2C38 interact with BsPYLs and BsSnRK2s in response to multiple abiotic stresses
Source: Front Plant Sci. 2022 Aug 11;13:963069. doi: 10.3389/fpls.2022.963069 (PMC9404246; doi:10.3389/fpls.2022.963069)

(A)

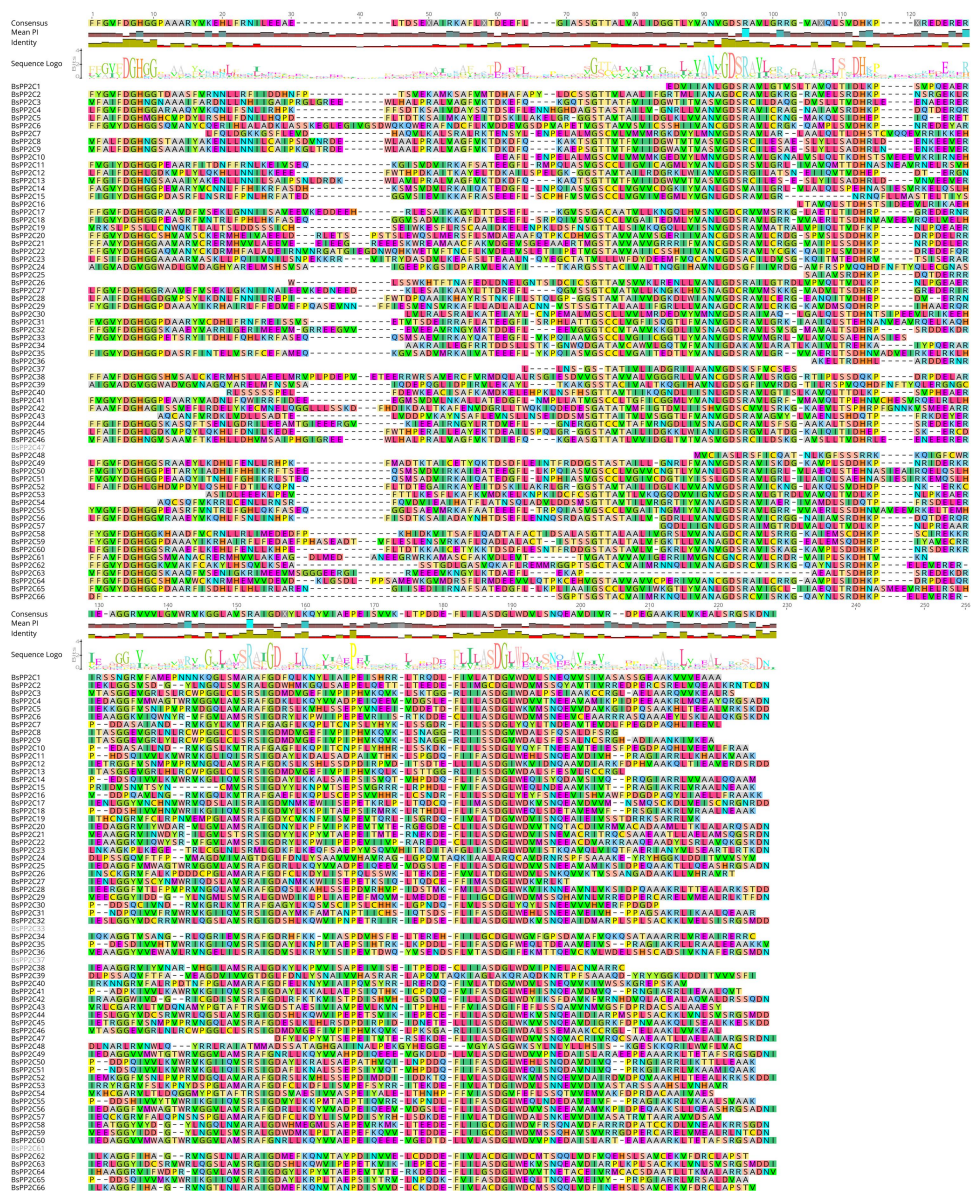

(B)

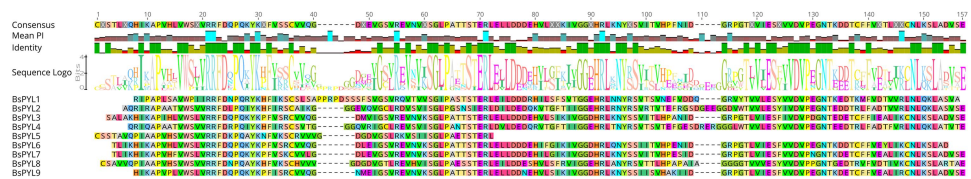

(C)

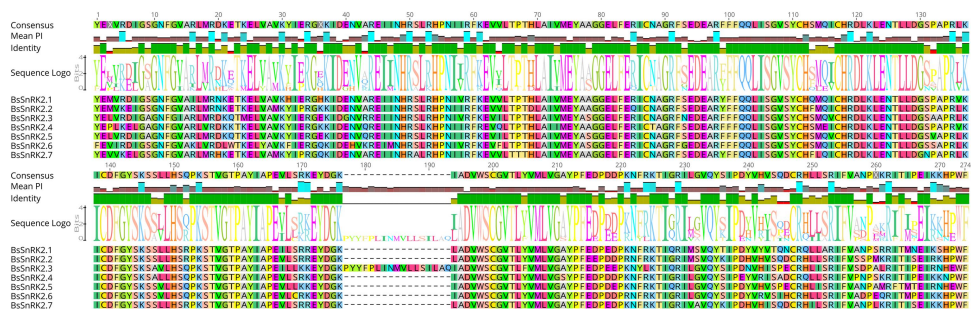

Figure S1

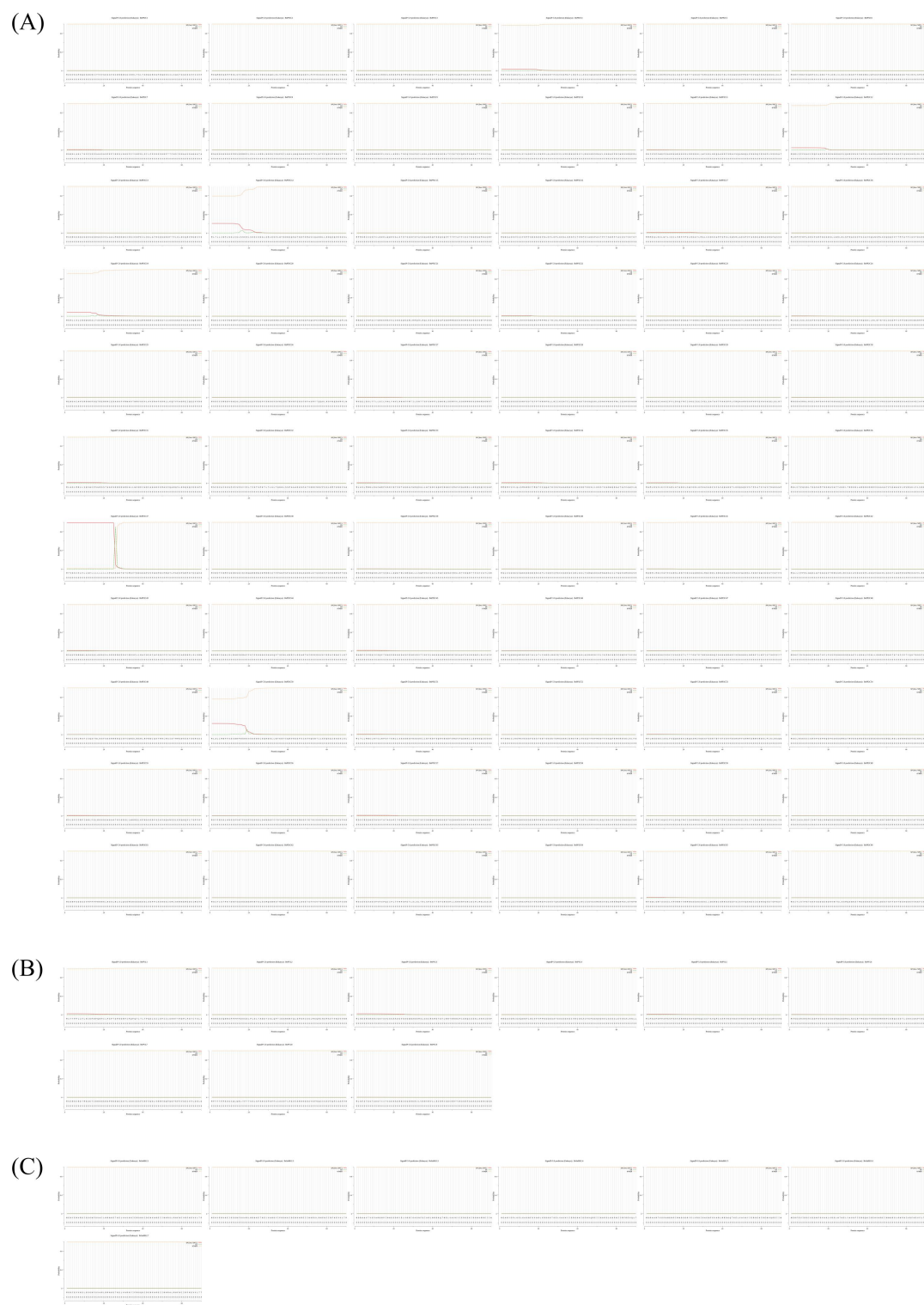

Figure S2

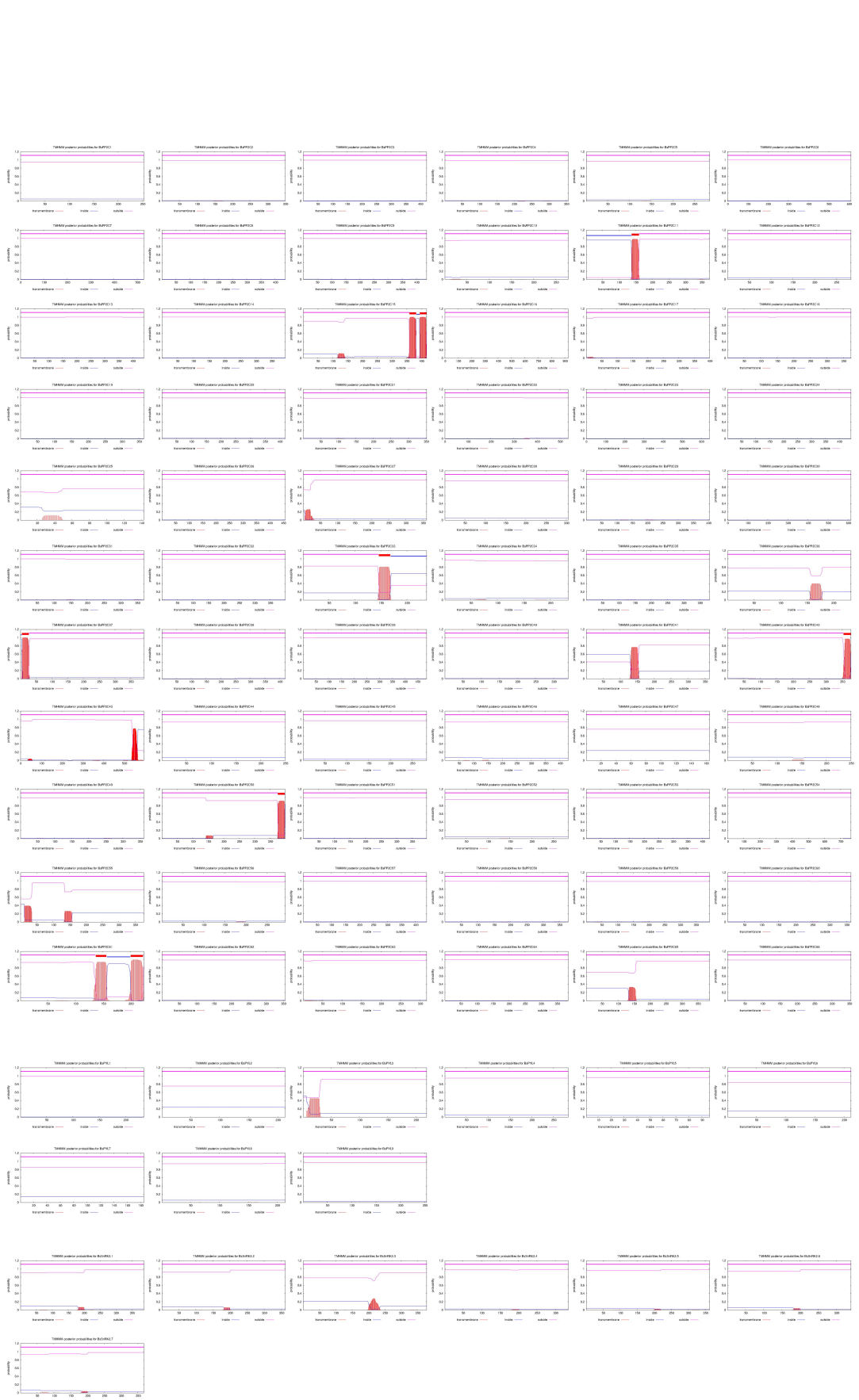

Figure S3

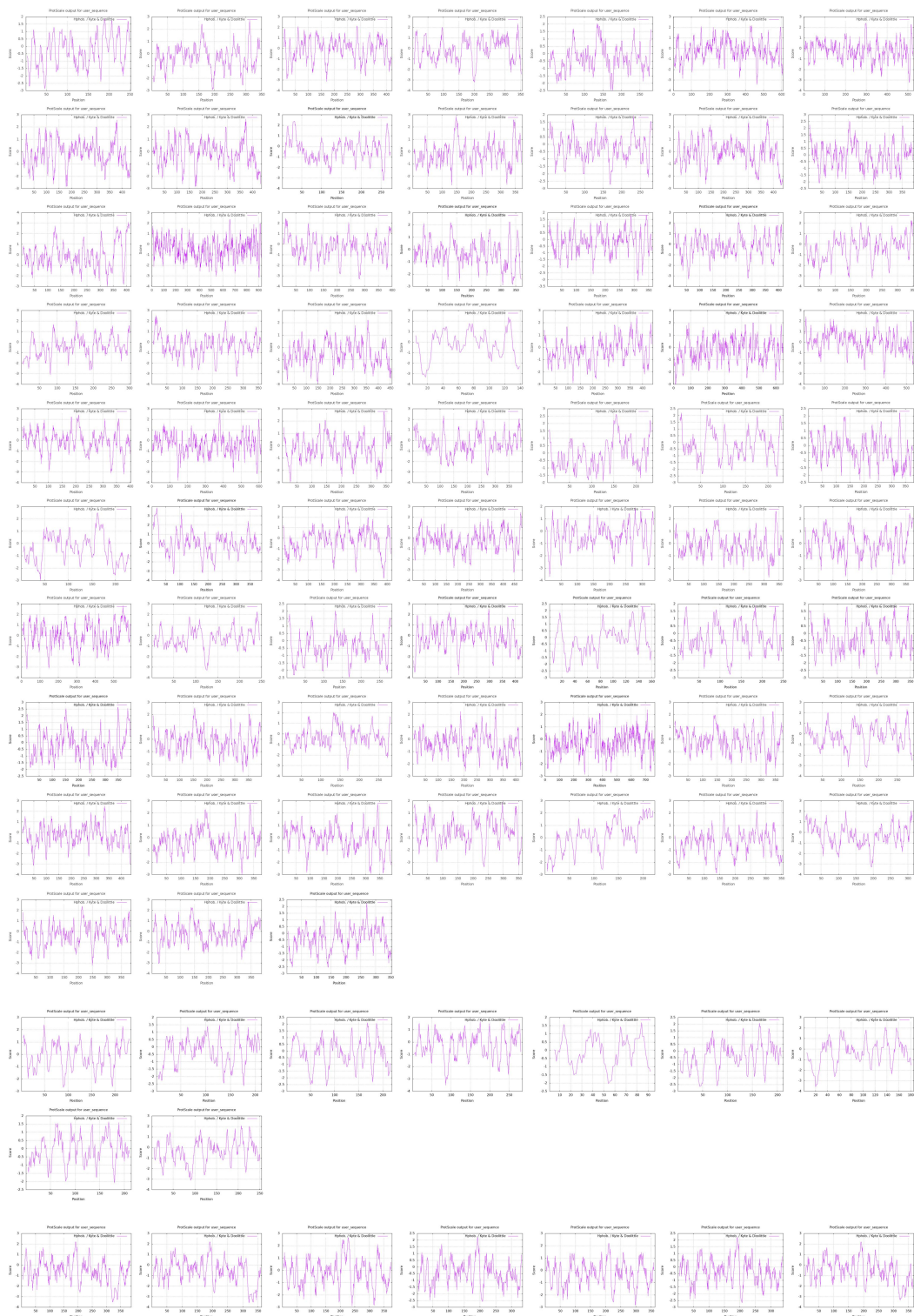

Figure S4

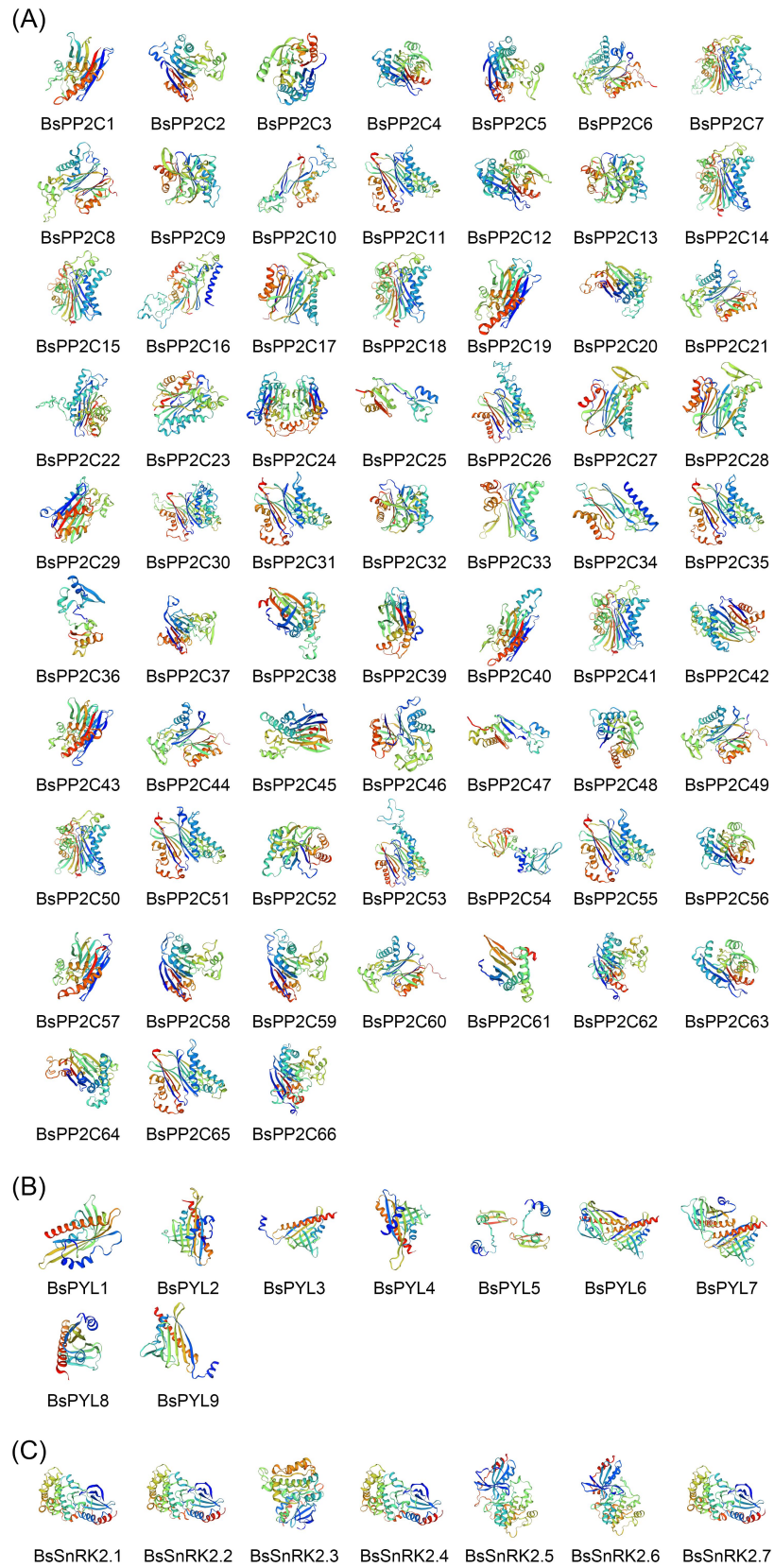

Figure S5

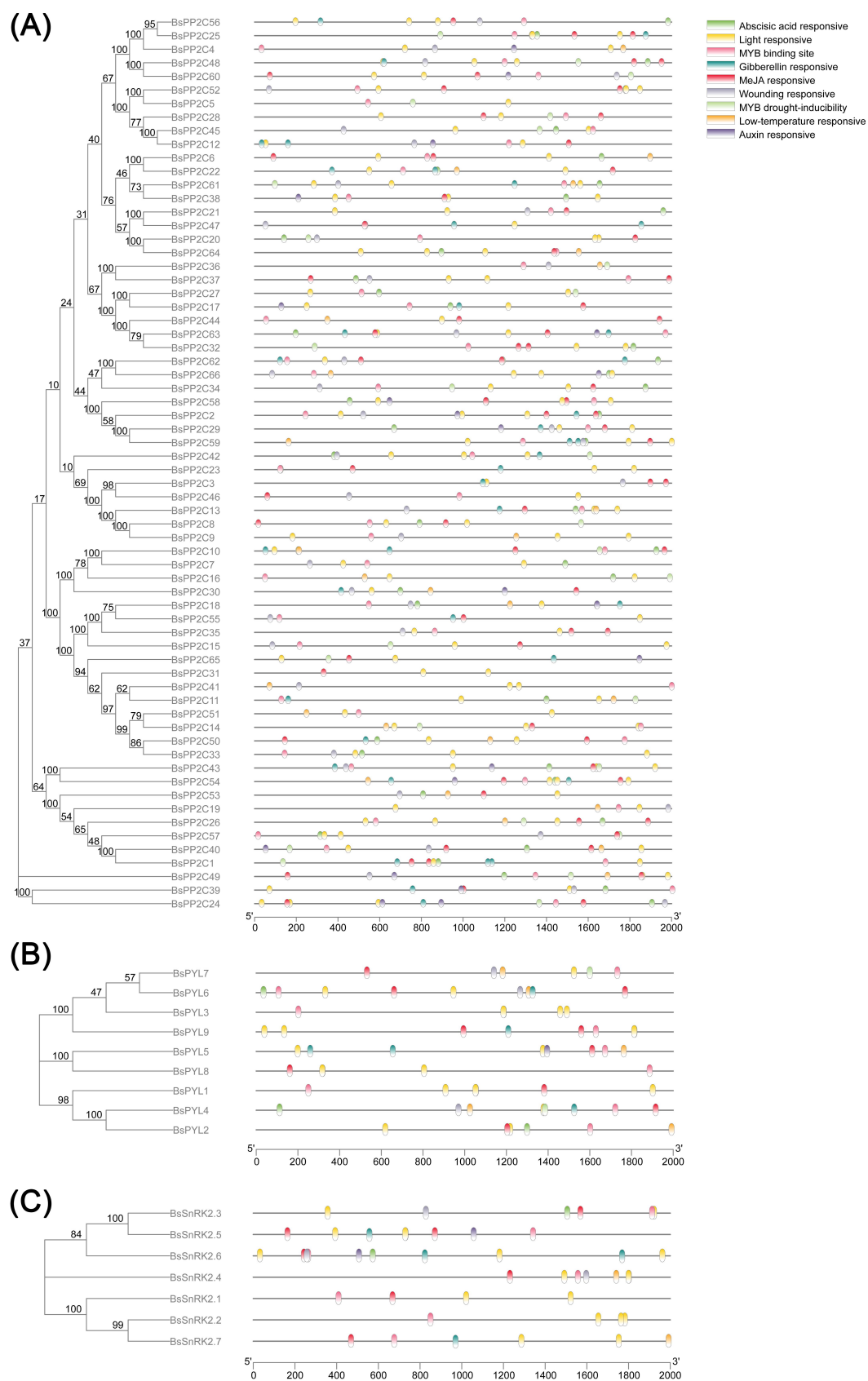

Figure S6

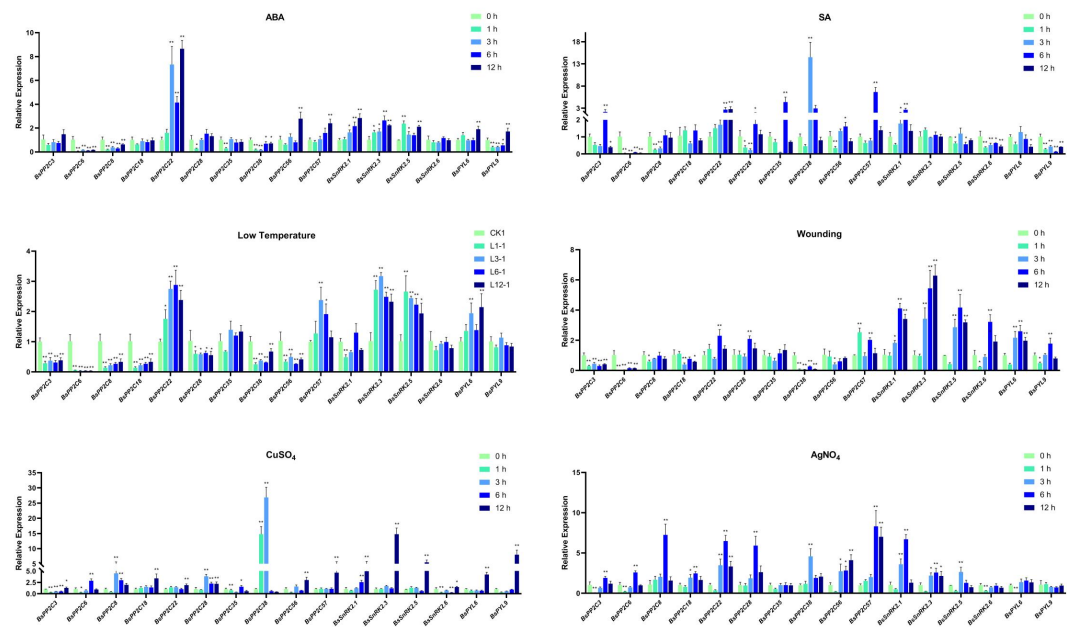

Figure S7

Supplement: Supplementary Figure 1 — The sequence logo of conserved motifs and the multiple sequence alignments of the BsPP2C (A), BsPYL (B), and BsSnRK2 (C) proteins conserved domain. [file Data_Sheet_2.pdf]
